# Free-viewing gaze patterns reveal a mood-congruency bias in MDD during an affective fMRI/eye-tracking task

**DOI:** 10.1007/s00406-023-01608-8

**Published:** 2023-04-23

**Authors:** Rui Sun, Julia Fietz, Mira Erhart, Dorothee Poehlchen, Lara Henco, Tanja M. Brückl, BeCOME study team, Elisabeth B. Binder, Angelika Erhardt, Susanne Lucae, Norma C. Grandi, Tamara Namendorf, Immanuel Elbau, Laura Leuchs, Anna Katharine Brem, Leonhard Schilbach, Sanja Ilić-Ćoćić, Julius Ziebula, Iven-Alex von Mücke-Heim, Yeho Kim, Julius Pape, Michael Czisch, Philipp G. Saemann, Victor I. Spoormaker

**Affiliations:** 1 Department of Translational Research in Psychiatry, Max Planck Institute of Psychiatry, Munich, Germany; 2 Department of Behavioral and Psychological Science, Zhejiang University, Hangzhou, China; 3 International Max Planck Research School for Translational Psychiatry (IMPRS-TP), Max Planck Institute of Psychiatry, Munich, Germany; 4 Max Planck Institute of Psychiatry, Munich, Germany

**Keywords:** Major depressive disorder, A mood-congruency bias, Emotional processing, Free-viewing, fMRI, Eye-tracking

## Abstract

Major depressive disorder (MDD) has been related to abnormal amygdala activity during emotional face processing. However, a recent large-scale study (*n* = 28,638) found no such correlation, which is probably due to the low precision of fMRI measurements. To address this issue, we used simultaneous fMRI and eye-tracking measurements during a commonly employed emotional face recognition task. Eye-tracking provide high-precision data, which can be used to enrich and potentially stabilize fMRI readouts. With the behavioral response, we additionally divided the active task period into a task-related and a free-viewing phase to explore the gaze patterns of MDD patients and healthy controls (HC) and compare their respective neural correlates. Our analysis showed that a mood-congruency attentional bias could be detected in MDD compared to healthy controls during the free-viewing phase but without parallel amygdala disruption. Moreover, the neural correlates of gaze patterns reflected more prefrontal fMRI activity in the free-viewing than the task-related phase. Taken together, spontaneous emotional processing in free viewing might lead to a more pronounced mood-congruency bias in MDD, which indicates that combined fMRI with eye-tracking measurement could be beneficial for our understanding of the underlying psychopathology of MDD in different emotional processing phases.

*Trial Registration*: The BeCOME study is registered on ClinicalTrials (gov: NCT03984084) by the Max Planck Institute of Psychiatry in Munich, Germany.

**Supplementary Information:**

The online version contains supplementary material available at 10.1007/s00406-023-01608-8.

## Introduction

The search for objective and clinically valuable biomarkers in psychiatry has been hampered by the purely symptomatic definition of mental disorders, e.g., according to the International Classification of Diseases (ICD-10, 1992) and the Diagnostic and Statistical Manual of Mental Disorders (DSM-5, 2013). For major depressive disorder (MDD), this has resulted in a combination of nine broadly varying symptoms, some of which are in opposing directions, which makes one specific biological underlying mechanism unlikely. Instead, the patchwork of varying symptoms warrants disentanglement into multiple measurable processes. To this end, several approaches have been proposed, with the Research Domain Criteria (RDoC) and Roadmap for Mental Health Research (ROAMER) initiatives as exemplary frameworks incorporating a variety of psychological constructs (e.g., positive and negative valence) that can be examined by cognitive/affective paradigms in a multimodal manner (e.g., at the physiological, neural, and behavioral levels) [1].

One commonly employed emotional processing task in clinical neuroscience and psychiatry is the emotional face-matching task, as first suggested by Hariri et al. [2]. This task is often incorporated into large-scale genetic and clinical psychiatric studies recruiting at least 1000 individuals (Human Connectome Project, from 2011; ENIGMA, from 2009; The Dunedin Study, from 1999; UK Biobank, from 2012) [3–6], as it reliably activates the amygdala [2, 7–11].

This task has also been incorporated frequently into studies focusing on MDD specifically, although evidence for group-average abnormalities in MDD has been inconsistent in small- or medium-sized studies [11–17]. Recently, a large-scale study including 28,638 participants’ data showed no correlation between amygdala activity (as induced by negative facial processing during the Hariri task) and depressive symptoms [18], impressively documenting that amygdala activation during emotional face processing does not represent a general biomarker of the clinical state of depression.

One possible reason for the lack of individual differences with respect to MDD could lie in the noisy nature of functional magnetic resonance imaging (fMRI) itself. It has been shown repeatedly that the test–retest reliability of fMRI activity is low in general [19–25]. This particularly applies to the emotional face-processing task with intra-class coefficients (ICC) of the amygdala ranging from − 0.02 to 0.16 [21]. Also, Hariri’s group has more recently observed low test–retest reliability of this task via meta-analysis and additional experimental evidence [7]. This could be due to the BOLD signal in the specific region being artifact prone [26], the general susceptibility of beta-values in a general linear model to non-neuronal events or fluctuations, the intrinsic biological property of the amygdala to habituate quickly, and fluctuations in attention that are typically not measured or considered. As test–retest reliability is the prerequisite for validity, such low reliability impedes the observation of any meaningful clinical correlations or between-group differences, as even an ICC of 0.16 suggests less than 2.56% shared variance between two subsequent measurements of the same task in the same individual.

One potential solution to improve the reliability of individual differences in clinical symptoms such as MDD is combining fMRI with simultaneous readouts such as eye tracking and pupillometry, which provide high-precision temporal and spatial information [27–29]. Such additional information may be utilized to make the fMRI readouts more specific and potentially more precise.

Previous work has shown differences in gaze behavior in MDD patients in related affective tasks such as the dot-probe task [30], attention disengagement [31], attention control [32], or mood induction [33]. In particular, two related meta-analyses have demonstrated that MDD patients compared to HC show moderate to large increases in the maintenance of gaze on dysphoric information (primarily face stimuli) and decreases in the maintenance of positive valence, reflecting what is referred to as “a mood-congruency bias” [34, 35]. This term denotes that the attentional behavior is systematically shifted toward stimuli containing depression-relevant information. To the best of our knowledge, differences between MDD and HC in gaze behavior during the commonly employed emotional face recognition task designed by Hariri et al. have not yet been studied.

In the context of MDD, a mood-congruency bias in this task might be challenging to detect because this gaze behavior is suppressed by the task instructions that usually ask participants to find the matching faces. Yet, in eye-tracking research, findings on mood-congruency gaze or attention have consistently been drawn from free-viewing tasks with the presentation of several emotional faces [35, 36]. In contrast to free-viewing gaze behavior primarily involving emotional processing, task-evoked gaze behavior could be additionally affected by executive control and decision-making. Consequently, emotional processing in task-evoked responses might be partly suppressed by other competing cognitive processing due to limited total processing resources. Besides, the neural response toward emotional stimuli is attenuated by high cognitive load [37]. Therefore, the mood-congruency bias drawn from free-viewing gaze behavior might not directly translate into task-evoked gaze behavior, whereas it might actually manifest itself more clearly in a free-viewing phase in the sense of an “unmasking.” Fortunately, for most studies employing this task, a typical version presents a stimulus (with three faces, two of which match in emotion) for 5–6 s, with a response typically requiring 1.5–3 s. This implies that every trial can be partitioned into a task-related and a free-viewing phase and that eye gaze behavior can be compared before and after the behavioral response.

To date, analyses of simultaneous multimodal measurements of combined fMRI with eye-tracking during the emotional face recognition task in MDD patients are lacking. Here, we sought to explore gaze behavior during Hariri et al.’s emotional face recognition task, examine the task-related vs. free-viewing phases separately and examine their respective neural correlates as measured by BOLD fMRI. To achieve this, we recorded fixation durations through an MRI-compatible eye-tracking system while participants performed the Hariri task in the MR scanner, following three aims. The first was to explore the mood-congruency bias in depression during the two phases in each trial. For this, we calculated dwell times by adding up single fixation durations on each face and trial phase (i.e., the task-related and free-viewing phase). We hypothesized that a mood-congruency attention bias in MDD would be manifested in the free-viewing but not the task-related phase. Second, we focused on contrasting amygdala responses from these two phases and hypothesized more robust amygdala activation in the free-viewing than the task-related phase. Third, we hypothesized that amygdala activation was associated with specific eye gaze patterns during the trial and operationalized this by adding the latter as parametric modulators of the fMRI response to the faces.

## Methods

### Participants

Our study cohort was recruited as a part of the study of the biological classification of mental disorders (the BeCOME study) at the Max Planck Institute of Psychiatry, Munich, Germany [38]. The BeCOME study aims to contribute to a biology-based taxonomy of affective, anxiety, and stress-related mental disorders for clinical applications. Exclusion criteria for the BeCOME study were: acute schizophrenia or psychotic symptoms, eating disorders, medication use within the last 2 months, as well as MRI contraindications. Participants took part in the computer-based International Diagnostic Interview (DIAX/M-CIDI) [39], which is used to assess symptoms and diagnose mental disorders according to DSM-IV.

﻿This study included 223 participants with measurements obtained between 2015 and October 2021. MDD patients were diagnosed as meeting the criteria within the past 12 months for the threshold of significant depression symptoms according to M-CIDI. HCs were defined as not fulfilling the criteria for any M-CIDI diagnosis in the lifetime. After removing 9 participants who were neither MDD patients nor HCs, the remaining 214 participants were included in the following analyses (see Supplementary Fig. S1 for the step-by-step exclusion procedure). All participants completed the Beck Depression Inventory (BDI-II) and the Montgomery–Asberg Depression Rating Scale (MADRS). Demographic information and scores of BDI-II and MADRS can be found in Table 1. The BeCOME study is in accordance with the Declaration of Helsinki and has been approved by the local ethics committee. All participants provided their written informed consent after the study had been fully explained, and were compensated for their participation.

**Table 1:** Demographic information and scores of BDI-II and MADRS in MDD and HCs

	*n*	% Female	Age (years, *M* ± SD)		BDI-II	MADRS
			Male	Female		
MDD	79	39.2	35.2 ± 14.1	33.3 ± 12.1	22.3 ± 10.0	18.7 ± 9.6
HC	135	31.1	33.4 ± 10.2	35.1 ± 13.3	4.6 ± 6.1	2.7 ± 5.7

MDD and HC represent the major depressive disorder patients and healthy controls; *M* and *SD* represent the mean score and standard deviation

### Face-matching task

**Fig. 1:**
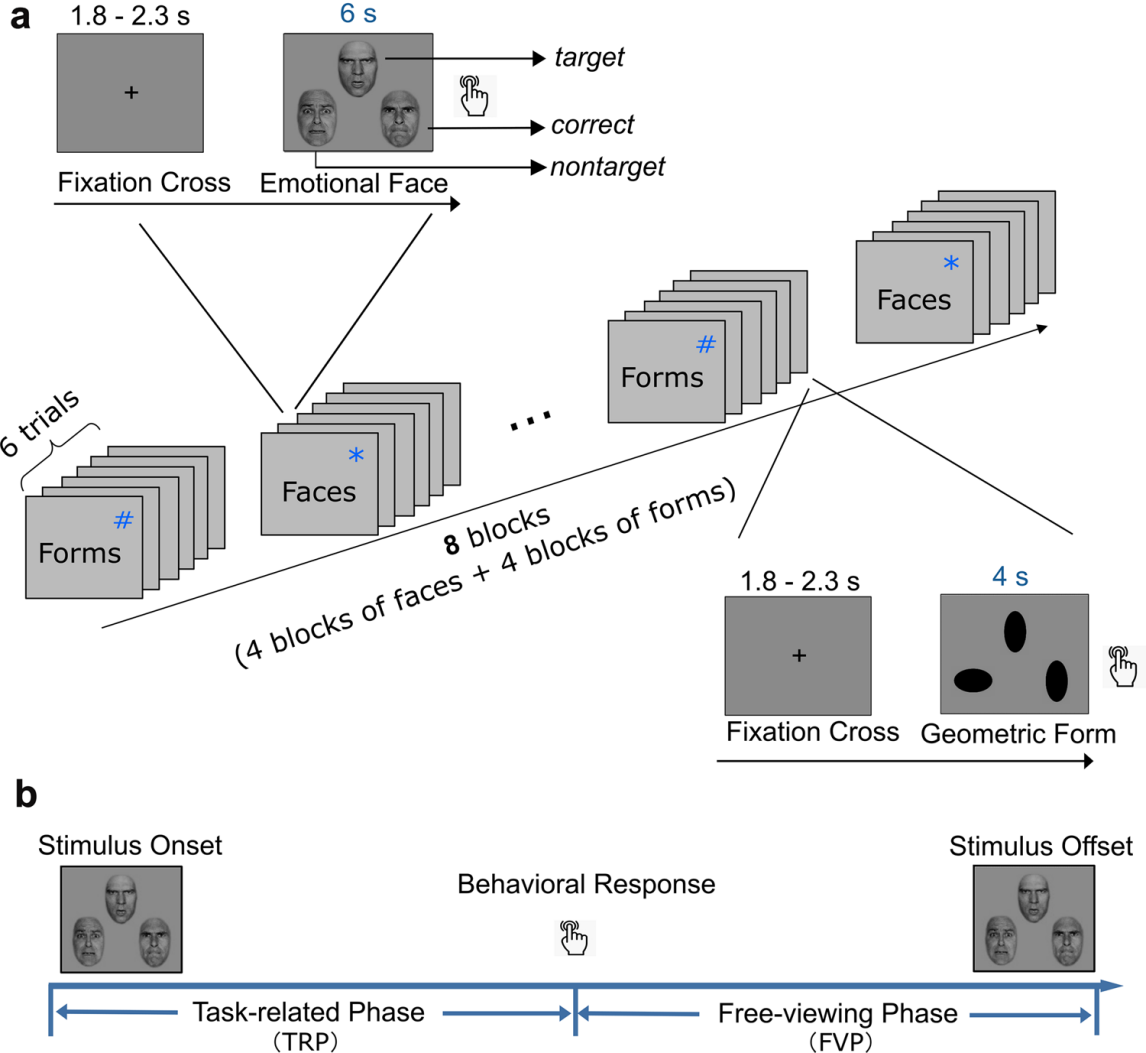
**a** Schematic representation of the experimental procedure. The task included eight blocks (four blocks for emotional faces (*) and the other four blocks for geometric forms (^#^)), each with six trials. In each trial, a stimulus with three objects (emotional faces presenting for 6 s and geometric forms presenting for 4 s) was displayed after a fixation cross (1.8–2.3 s). During the presentation of the stimulus, participants were instructed to press the corresponding button according to emotional expression or shape. **b** Schematic illustration of the task-related phase (TRP) and the free-viewing phase (FVP). The two phases were allocated based on the response timing in a trial with 6 s (emotion recognition) or 4 s (shape recognition). TRP was defined as the time from the start of the stimulus display to the behavioral response (button press), while FVP was defined as the time from the behavioral response to the end of the trial (stimulus offset). The facial stimuli are reproduced with permission from the Paul Ekman Group

We employed a face-matching task with a mixed block/event-related design [2]. This task presented four blocks of emotional faces and four blocks of geometric forms in an interleaved way, each consisting of six pseudo-randomized trials, with a total duration of 9:35 min for the whole task. During each trial presentation, one target object and two additional objects were separately presented on the middle top, left bottom, and right bottom of the screen (see Fig. 1a). During either the face trial lasting for 6 s or the form trial lasting for 4 s, the (top) target needed to be matched according to its facial expression (for faces) or shape (for forms) to one of the two objects at the bottom. Thirty-six female and thirty-six male faces were derived from the Picture of Facial Affect database [40], adjusted to the same size and luminosity, with the hairstyle removed. The emotional faces were portrayed as either happy, neutral, sad, angry, or fearful facial emotions. They were combined into six types of pairings: happy–sad, happy–angry, happy–fearful, sad–neutral, sad–angry, and angry–fearful pairs. The whole stimulus with three objects was 1024 × 768 pixels, with 160 × 200 pixels for each object. The experimental stimuli were displayed with Presentation Software version 18.01 (Neurobehavioral Systems Inc., Berkeley, CA, U.S.A.).

### Experimental procedure

Participants who met the criteria performed the face-matching task on the second day of the study. Before starting the formal experiment, participants received instructions about the task and practiced how to respond to the stimuli outside the scanner. In each trial, after a 1.8–2.3 s fixation cross, participants should choose one of the two bottom objects (see Fig. 1a). The training stage would stop until participants were fully familiar with the whole procedure of the experiment performed in the scanner.

In the formal experiment, participants then viewed stimuli inside the MR scanner by a mirror with a 45° angle above the head coil. The mirror reflected the monitor’s screen which was positioned approximately 2 m behind the end of the scanner bore.

### Stimulus pairs and phases

As for experimental manipulation, the stimuli were classified according to the following criteria, and each classification was as within-subject variables. First, we used stimulus type: emotional face vs. geometric form. Then all emotional faces can be divided into positive–negative pairs and negative–negative pairs based on the emotional valences involved in each face pair. We used all positive–negative pairs (i.e., happy-sad, happy-angry, and happy-fearful pairs) to examine the negativity bias, which indicated biased attention to all negative faces. Moreover, to examine the mood-congruency bias (for mood-congruent stimuli specifically, in this case, sad faces), we compared the happy–sad faces with the other positive–negative pairs (e.g., fearful, angry, and the positive emotion of always being happy).

Additionally, the processing phase within a trial was also analyzed by defining a within-subject factor, with one level denoting the task-related phase (TRP) and the other level denoting the free-viewing phase (FVP), which differed according to the response timing. TRP was defined as the time from the start of the stimulus display to the button press, while FVP was defined as the time from the button press to the end of the trial (see Fig. 1b).

### Behavioral data

Mean response times (RT) and mean accuracy rates were calculated within and across conditions. An accurate response was defined as only one correct answer (response) in a single trial, while no response or multiple responses in a trial were regarded as inaccurate. In case the task difficulty confused the results, we removed 24 participants with an accuracy rate lower than 75% (see S1.1 Group differences in accuracy rates in the supplementary material). Thus, 190 participants were included in the behavioral analyses (see Supplementary Fig. S1 for the exclusion procedure and Supplementary Table S1 for demographic information and descriptive statistics of behavioral values).

### Eye-tracking data acquisition and processing

An MRI-compatible eye-tracking device (EyeLink 1000 plus; SR Research Ltd., Osgoode, ON, Canada) was adopted to record the pupil and the eye movements of the right eye with a sampling rate of 250 Hz. A standard 9-point calibration was applied to calibrate eye gaze position. The eye-tracking data were extracted and analyzed in MATLAB (version 2021a, MathWorks, Natick, MA, USA). For fixation analyses, a trial was included if over 75% of fixations were located in the regions of interest (ROI). We removed 20 participants as they had more than 20% invalid trials of the whole task. Thus, 170 individuals’ data were included in the eye-tracking analyses (see Supplementary Fig. S1 for the exclusion procedure and Supplementary Table S2 for demographic information and descriptive statistics of gaze values).

Before computing the values of eye-tracking readouts, three ROIs were ensured by ellipses of the same shape and size, with 194 pixels in the short and 229.5 pixels in the long radius. The central points of the three ROIs were (510, 246.5) for the (top) target, (198, 526.5) for the left one, and (815, 535.5) for the right one.

To measure attentional maintenance, we analyzed dwell times, defined as the total fixation time during each phase (i.e., the task-related or free-viewing phase). We computed the proportion of dwell times (*pDT*) on different ROIs: dwell times (*DT*) on one ROI (i.e., top, right, or left) were divided by the total dwell times (*total DT*) on all three ROIs.

1 $$pDT = \frac{DT}{{total DT }}$$

To get a comprehensive value per trial, which was used in a parametric modulation in GLMs, we computed the differential *pDT* between the faces with the same emotional expression (*pDT*_target_ and *pDT*_correct_) and the face with the distinct emotion (*pDT*_nontarget_), separately for the task-related and free-viewing phase, reflecting the preference of task-related attention (*PTRA*). It is worth noting that analyses of the mood-congruency or negativity attention bias were conducted for positive–negative pairs. A higher negative attention bias would be indicated in these two patterns: (1) a higher *PTRA* in trials with the negative target (top) faces; (2) a lower *PTRA* in trials with positive target faces. Therefore, a negative attention bias was defined as *PTRA* values in trials with negative target faces or opposites of *PTRA* values in trials with positive target faces. *PTRA* values were calculated as follows:

2 $$PTRA = { }pDT_{{{\text{target}}}} + pDT_{{{\text{correct}}}} - pDT_{{{\text{nontarget}}}} { }$$

### Statistical analysis of eye-tracking data

First, to test whether there were differences in the effect of stimulus type on *PTRA* between the two groups, a two-factorial ANOVA (factor *group*, two levels: MDD vs. HC; factor *stimulus*, two levels: emotional face vs. geometric form) was conducted.

Then for testing the negativity bias, only three kinds of happy–negative pairs were included in the analysis. Again, a two-factorial ANOVA (factor *group*, two levels: MDD vs. HC; factor *specific-valenced pair*, three levels: happy–sad vs. happy–angry vs. happy–fearful) was adopted.

Last, for testing the mood-congruency attention bias, three kinds of happy–negative pairs were adjusted to two types of emotional pairs, consisting of happy–sad and happy–other negative (i.e., happy-angry and happy-fearful) pairs. A two-factorial ANOVA (factor *group*, two levels: MDD vs. HC; factor *specific-valenced pair*, two levels: happy–sad vs. happy–angry–fearful) was adopted.

All of the above analyses of *PTRA* were conducted separately for the task-related and the free-viewing phase. The significance level *alpha* for all behavioral and eye-tracking analyses was set at 0.05, with Bonferroni correction for multiple contrasts in the post hoc tests.

### fMRI data acquisition and preprocessing

The fMRI data were acquired from a 3 Tesla MRI Scanner (Discovery MR750, GE, Milwaukee, WI). A 32-channel head coil was used covering 40 slices (AC-PC orientation of the slices, 3 mm slice thickness, 0.5 mm slice gap, resulting voxel size 2.5 × 2.5 × 3.5 mm^3^, 96 × 96 matrix, in-plane field of view 24 × 24 cm^2^, echo planar imaging [EPI], TR 2.5 s, TE 30 ms, acceleration factor 2). A total of 230 image volumes were acquired in the Hariri task, of which the first 4 volumes were deleted due to non-steady-state effects.

Data were preprocessed by Statistical Parametric Mapping (SPM12, Wellcome Centre for Human Neuroimaging, London, UK, http://www.fil.ion.ucl.ac.uk/SPM) in a MATLAB (version 2021a, MathWorks) environment. Data preprocessing consisted of (a) realignment with rigid body motion correction, using the first image of the scanning as a reference, and an extra FSL-based rigid body motion to calculate differences on root-mean-squared intensity between volumes; (b) slice time correction taking the bottom-up acquisition interleaved way; (c) co-registration of the temporal series by a specific contrast-rich T2-weighted EPI image (TR = 10 s); (d) segmentation of the specific image utilizing unified segmentation (REF) in SPM to gain gray matter (GM), white matter (WM), and cerebrospinal fluid (CSF) probability map, (e) spatial normalization to MNI space using the iterative DARTEL technique with IXI templates (driven by GM and WM), (f) interpolation of the normalized image to 2 × 2 × 2 mm^3^ resolution, and (g) spatial smoothing by a Gaussian Kernel (full width at half maximum: 6 × 6 × 6 mm^3^). Denoising was performed in each first-level GLM by adding the following nuisance covariates: (i) three components of WM and CSF (based on in Step (d)) and (ii) their temporal derivatives. The threshold for exclusion was set at a 2 mm shift between two sequential volumes and 1° rotation in any direction. Eight participants for technical reasons and eleven for exceeding the movement threshold. Thus, 151 participants were included in the fMRI analyses (see Supplementary Fig. S1 for the exclusion procedure and Supplementary Table S3 for demographic information).

### First-level analysis

First-level and second-level analyses were also performed in SPM12. For the first-level general linear model (GLM), we dissected the trial into the task-related and free-viewing phases to compare the brain activity in separate phases. To minimize the effect of intrinsic temporal collinearity of these regressors [41], we created separate first-level GLMs for each phase.

First, to examine the effect of stimulus type on amygdala activation, we created two first-level GLMs with the block-related design separately for each phase. The T contrasts of stimulus type were as follows: [+ 1 − 1] representing the differential contrast “emotional faces [+ 1] > geometric forms [− 1]”, and [+ 1] representing “emotional faces ([+ 1])”, tested against an average background.

Then to examine neural correlates of *PTRA* obtained from emotional faces, we created two first-level GLMs with the event-related design for each phase. *PTRA*s from each stimulus and phase were entered as parametric modulators into the first-level GLMs. The T contrast of *PTRA*s of stimuli was as follows: “*PTRA* of emotional faces ([+ 1])”, testing for a positive correlation between the respective *PTRA* values and emotional faces.

### Second-level random effect analysis

Contrast maps of first-level T contrasts, as mentioned above, were entered into the second-level models: first, one-sample *t *tests for both phases using contrast images of all participants were conducted for stimulus types, with contrasts (all participants [+ 1] > [0] and all participants [− 1] < [0]).

Second, to explore the neural correlates of distinct emotional processing phases, we compared the brain activity of two different phases (i.e., the task-related and free-viewing phase) and conducted paired *t *tests using all participants’ data for emotional faces and *PTRA* of emotional faces, both with contrasts (TRP [+ 1] > FVP [− 1] and TRP [− 1] < FVP [+ 1]).

Finally, to test for differences between the MDD and healthy group (HC), two-sample *t* tests for both phases using contrast images of first-level analyses (the task regressors and the parametric modulation with *PTRA* in the task-related and free-viewing phase) were conducted with contrasts (MDD [+ 1] > HC [− 1] and MDD [− 1] < HC [+ 1]).

The significance threshold was set at a voxelwise *p*_FWE_ < 0.05 and *k* > 30. Labeling of the relevant clusters of activity was performed with the automated anatomical labeling (AAL) atlas (AAL 2 toolbox) [42].

## Results

### Behavioral results

**Fig. 2:**
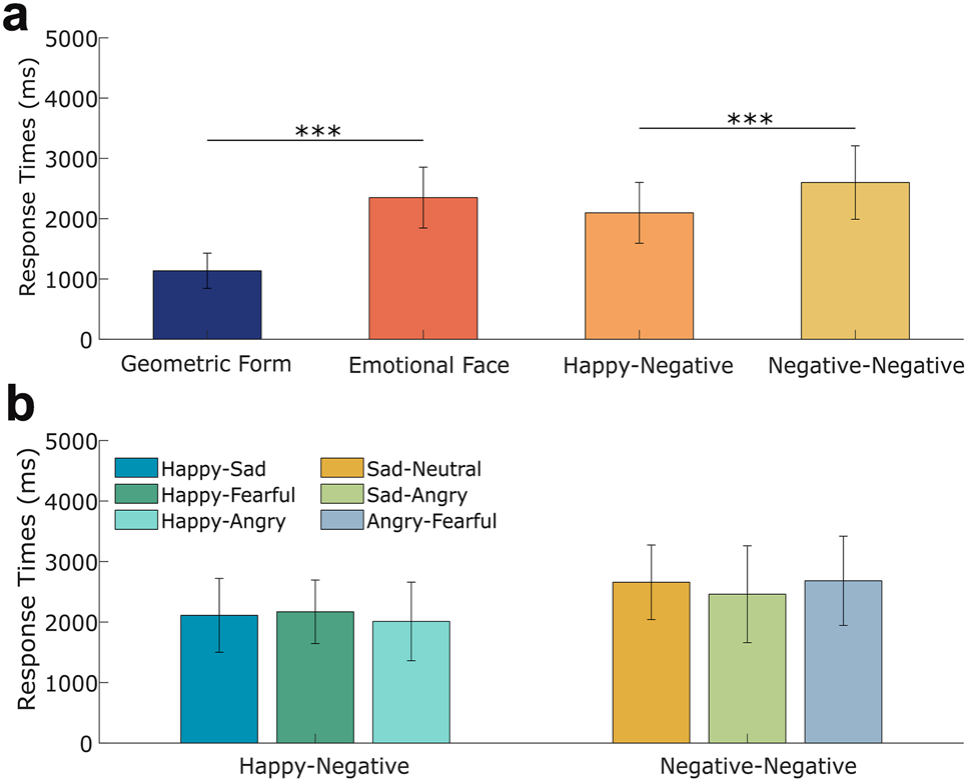
Response times (RT) for **a** different stimulus types and emotional pairs (i.e., happy–negative and negative–negative pairs); **b** the specific emotional stimulus pairings. Error bars denote standard errors. ***p < 0.001. *ms* milliseconds

Our RT analyses found no group differences in RTs between MDD patients and HCs (*F*_(1,188)_ = 0.31, *p* = 0.57). However, there was a main effect of stimulus type (*F*_(1,188)_ = 1885.17, *p* < 0.001, *η*^2^_p_ = 0.91) with longer RTs for emotional faces than geometric forms (*t*_(189)_ = 43.42, *p* < 0.001, Cohen’s *d* = 3.16, see Fig. 2a).

Then emotional face pairs were divided into positive–negative pairs (composed of happy–sad, happy–angry, and happy–fearful pairs) and negative–negative pairs (composed of sad–neutral, sad–angry, and angry–fearful pairs). We conducted a 2 groups × 2 emotional pairs ANOVA, which again found no group differences (*F*_(1,188)_ = 0.25, *p* = 0.62). We found a main effect of emotional pair (*F*_(1,188)_ = 514.68, *p* < 0.001, *η*^2^_p_ = 0.73) with longer RTs for negative–negative pairs than positive–negative pairs (*t*_(189)_ = 22.69, *p* < 0.001, Cohen’s *d* = 1.22, see Fig. 2a). Figure 2b shows the RTs of all emotion combinations.

Last, happy–negative pairs were further divided into happy–sad and happy–angry–fearful pairs. We conducted a 2 groups × 2 specific-valenced pairs ANOVA, which showed that there was no main effect of group (*F*_(1,188)_ = 0.37, *p* = 0.55) and no interaction between group and specific-valenced pair (*F*_(1,188)_ = 0.76, *p* = 0.39).

### Eye-tracking results

The results of *PTRA* were analyzed separately for the task-related and the free-viewing phase. We first tested the group differences in *PTRA* across stimulus types (emotional face vs. geometric form). In the task-related phase, the ANOVA revealed a significant main effect of stimulus type (*F*_(1,168)_ = 309.12, *p* < 0.001, *η*^2^_p_ = 0.58), indicating a higher *PTRA* for emotional faces than geometric forms (*t*_(169)_ = 20.70, *p* < 0.001, Cohen’s *d* = 1.34) but no effect of group or group-by-stimulus (*F*s < 0.44, *p*s > 0.50). In the free-viewing phase, no significant main and interaction were detected (*F*s < 1.64, *p*s > 0.20).

Then the ANOVAs regarding the negativity bias showed no significant group effects in either phase (*F*s < 0.02, *p*s > 0.90), indicative of no negativity bias in MDD. There was an interaction between group and emotional pair (happy–sad vs. happy–fearful vs. happy–angry) in the free-viewing phase (*F*_(2,336)_ = 3.44, *p* = 0.03, *η*^2^_p_ = 0.02), with the post hoc test pointing to a lower *PTRA* for happy–sad pairs compared with happy–angry and happy–fearful pairs in MDD (*t*s > 3.42, *p*s < 0.01, Cohen’s *d* > 0.33), meaning that MDD patients had more attentional preferences for sad faces than angry and fearful faces. However, no such interaction was detected in the task-related phase (*F*_(2,336)_ = 2.47, *p* = 0.09).

**Fig. 3:**
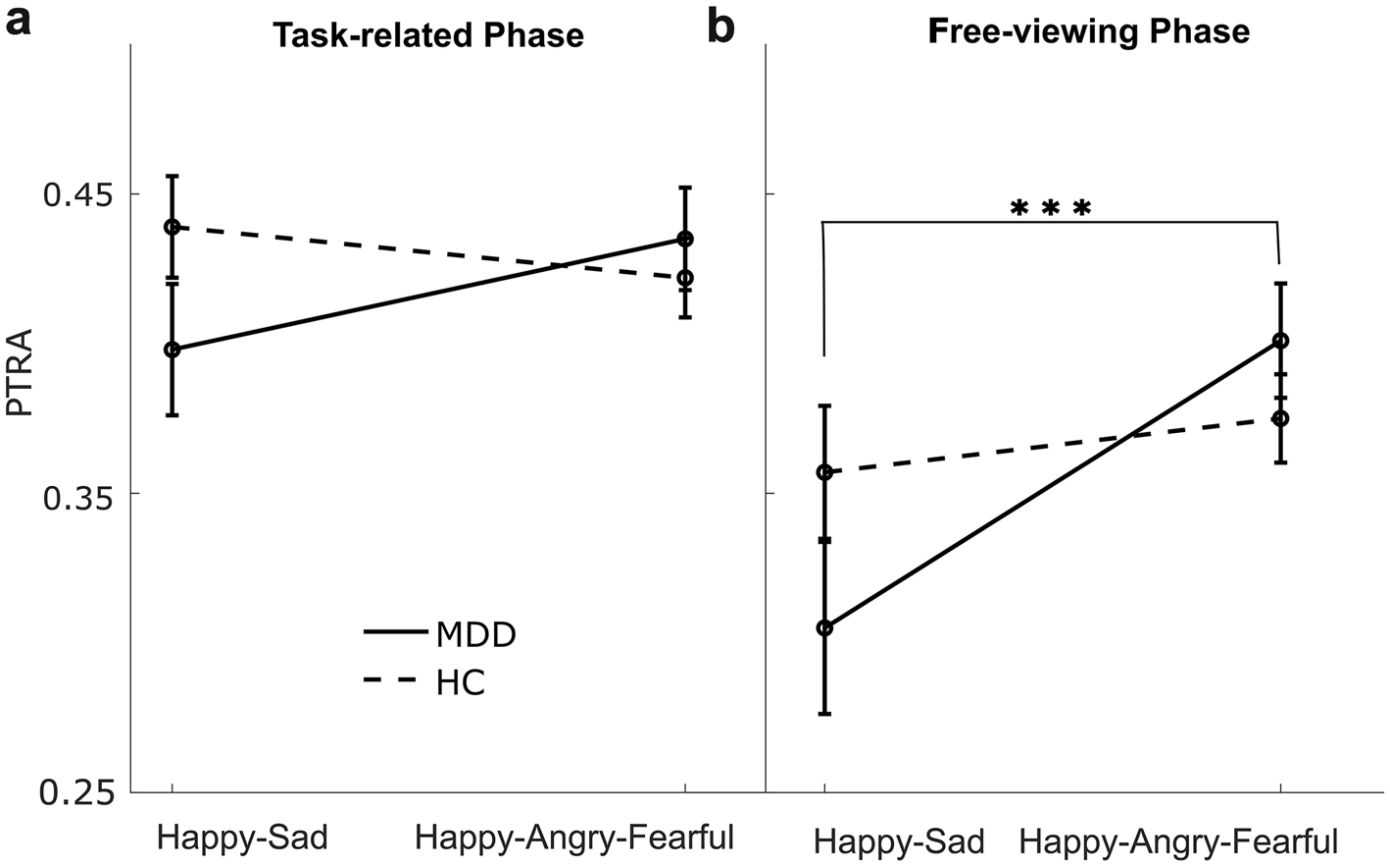
Interactions between group and the specific-valenced pair in the task-related phase (**a**) and the free-viewing phase (**b**). In the free-viewing phase, MDD patients had lower *PTRA* for happy–sad pairs than other mixed-emotional pairs, indicating that they had more attentional preferences for sad faces than other negative ones. Error bars denote standard errors. ***p < 0.001. *MDD* major depressive disorder, *HC* healthy control

Last, the ANOVAs regarding a mood-congruency bias showed there was an interaction between group and specific-valenced pair (happy–sad vs. happy–angry–fearful) in the free-viewing phase (*F*_(1,168)_ = 6.42, *p* = 0.01, *η*^2^_p_ = 0.03). The post hoc test showed that MDD patients had a lower *PTRA* for happy–sad pairs compared with happy–angry–fearful pairs (*t*_(64)_ = − 3.95, *p* < 0.001, Cohen’s *d* = − 0.46, see Fig. 3b), meaning that they had more attentional preferences for sad faces than other negative faces, which indicated a mood-congruency bias in MDD. However, in the task-related phase, the ANOVA only revealed a trend for an interaction (*F*_(1,168)_ = 3.40, *p* = 0.06) with no significant contrasts across groups and specific-valenced pairs for the post hoc test (*t*s < 1.70, *p*s > 0.55, see Fig. 3a).

### fMRI results

#### Neural correlates of experimental conditions

**Fig. 4:**
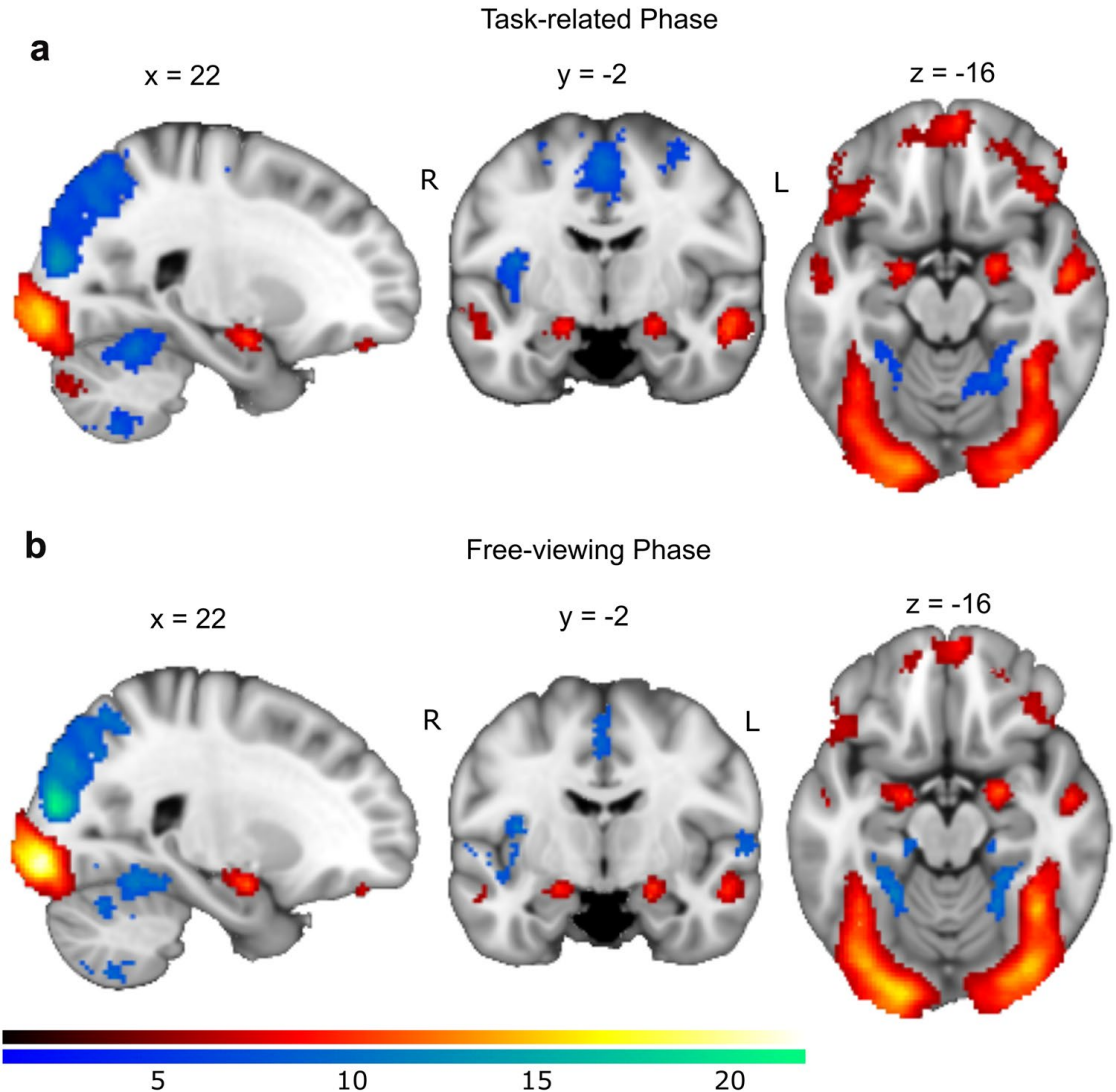
Brain activity between stimulus types analyzed separately for the task-related phase (**a**) and the free-viewing phase (**b**). Hot colors indicate a higher BOLD response to emotional faces compared with geometric forms; cold colors refer to the inverse contrast. The background template is a mean T1-weighted image with DARTEL spatial normalization. L = left, R = right. Color bars indicate *T* values. *p*_FWE_ < 0.05, *k* > 30

Second-level analyses showed that both groups shared significant whole-brain corrected activation clusters in the bilateral amygdala in the task-related phase (right: *x* = 22, *y* = − 4, *z* = − 16, *t* = 10.57, *p*_FWE_ < 0.001; left: *x* = − 18, *y* = − 6, *z* = − 16, *t* = 9.91, *p*_FWE_ < 0.001) and in the free-viewing phase (right: *x* = 22, *y* = − 4, *z* = − 16, *t* = 11.81, *p*_FWE_ < 0.001; left: *x* = − 18, *y* = − 6, *z* = − 16, *t* = 10.91, *p*_FWE_ < 0.001), when emotional faces were compared to geometric forms (see Fig. 4 and Supplementary Tables S4 and S5). Yet, we found no significant differences between MDD patients and HCs.

#### Comparison of amygdala activation in each trial phase (free viewing vs. task related)

**Fig. 5:**
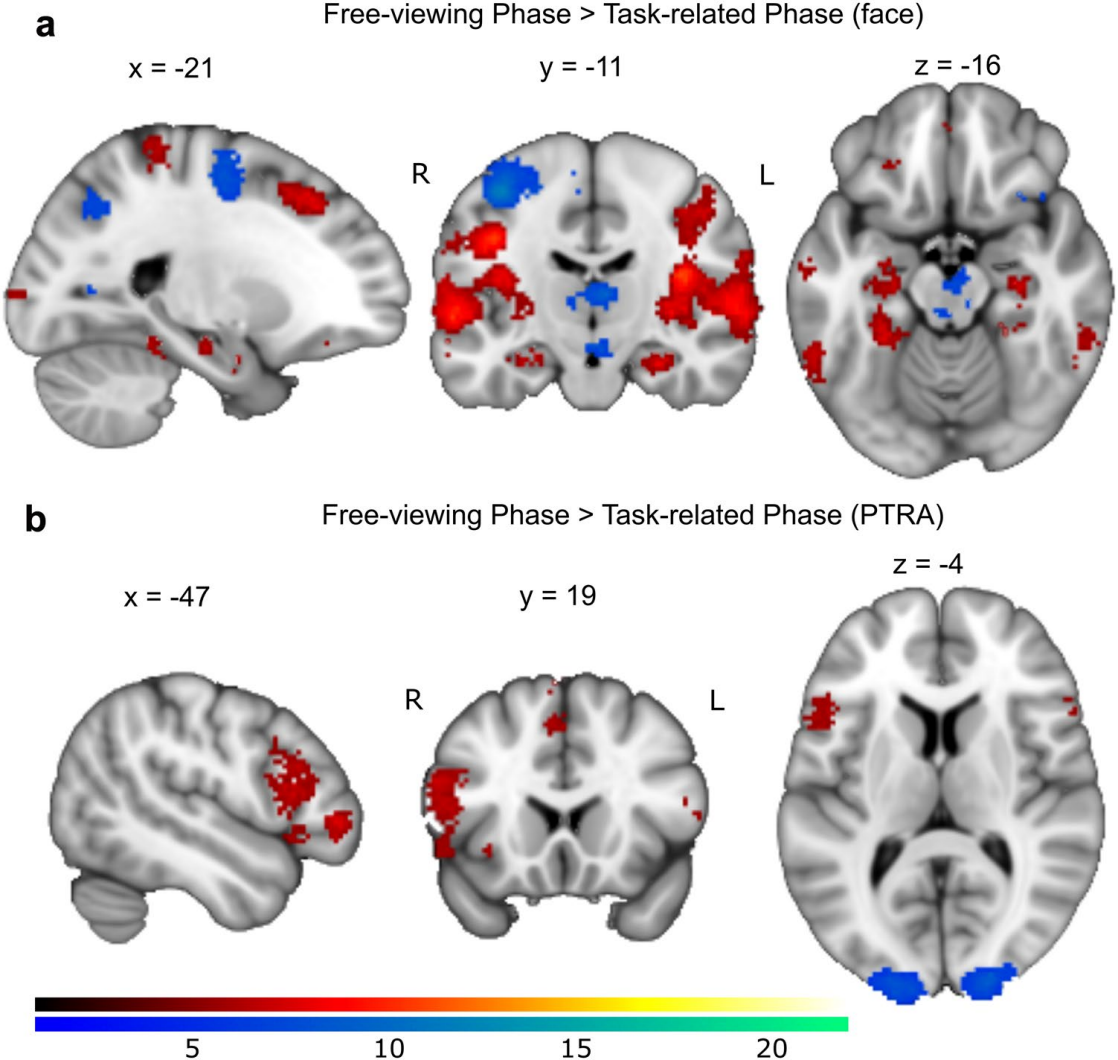
Brain activation (**a**) and neural correlates of *PTRA* (**b**) for each emotional face. Hot (cold) colors refer to more (less) activation in the free-viewing phrase compared to the task-related phase. The background template is a mean T1-weighted image with DARTEL spatial normalization. L = left, R = right. Color bars indicate *T* values. *p*_FWE_ < 0.05, *k* > 30

The second-level GLM showed significant amygdala activation to all emotional faces in the free-viewing phase compared to the task-related phase across individuals (see Fig. 5a and Supplementary Table S6), which was accompanied by activity in other brain regions such as the bilateral temporal gyri (middle and superior), right supplementary motor area (SMA), bilateral paracentral gyri, bilateral hippocampus, left fusiform and bilateral occipital gyri (middle and superior). In contrast, the response to emotional faces was higher in the task-related phase in the bilateral SMA, bilateral insula, bilateral inferior triangular-frontal gyri, bilateral anterior and middle cingulate gyri, left precentral gyrus, and right inferior orbital-frontal gyrus than the free-viewing phase.

#### Comparison of neural correlates of *PTRA* in each trial phase (free viewing vs. task related)

The second-level GLM showed that in the comparison of the free-viewing phase to the task-related phase, there were significant clusters of activity related to *PTRA* of emotional faces in bilateral inferior orbital-frontal gyri, left inferior operculum- and triangular-frontal gyri, bilateral superior medial-frontal gyri, left SMA and right cerebellum (crus II) (see Fig. 5b and Supplementary Table S7). In contrast, clusters of activity were higher in the task-related phase in bilateral calcarine, bilateral lingual gyri, bilateral occipital gyri (inferior, middle, and superior), and bilateral fusiform compared to the free-viewing phase. This indicates a shift from visual areas in the task-related phase to more lateral prefrontal activity in the free-viewing phase.

#### Group differences in neural correlates of *PTRA*

We neither observed any clusters of activity at the significance level of voxelwise *p*_FWE_ < 0.05 nor at cluster-wise *p*_FWE_ < 0.05 (with the statistical maps being collected at uncorrected *p* < 0.001, *k* > 50) in the group comparisons of the two contrasts: the parametric modulations of *PTRA* in the task-related and free-viewing phase. When we lowered the collection threshold to uncorrected *p* < 0.005, *k* > 50, we observed significant clusters of activity at cluster-wise *p*_FWE_ < 0.05 during the free-viewing phase. The comparison of MDD patients > HCs revealed clusters of activity in the bilateral middle and posterior cingulate cortex, the left middle occipital, and calcarine gyri. The reverse contrast revealed clusters of activity in the bilateral SMA extending into the left superior frontal gyrus (see Supplementary Fig. S3b and Table S9 and see Supplementary Fig. S3a and Table S8 for the statistical parametric map and descriptive information of significant clusters in the task-related phase). The neural correlates of each phase, per group, are provided in Supplementary Figs. S4–S5 and Tables S10–S13.

## Discussion

In this study, we studied gaze patterns and neural responses during an emotional face-matching task in MDD patients and healthy controls, focusing on a careful separation between a task-related and a free-viewing phase of trials. More specifically, our combined fMRI/eye-tracking setup allowed us to study attentional biases in the form of dwell times between distinct facial expressions during a single trial. Eventually, we combined both sources of information by weighting the fMRI analyses of stimulus-specific effects by gaze information.

In line with our first hypothesis, we observed a robust group difference in gaze patterns on relevant emotional faces in the free-viewing phase, reflecting a mood-congruency bias in MDD. This result is in accordance with previous work reporting a mood-congruency bias in depressed individuals [33, 36, 43–45], validating the setup as a whole theoretically, as gaze patterns may be distorted or more challenging to measure in the MRI environment. Indeed, a mood-congruency bias was detected, as reflected in three main findings. First, we examined group differences in gaze behavior of viewing happy–sad face pairs in both phases. We observed an interaction of group and emotion in the free-viewing phase, showing that MDD patients had less biased attention to happy faces than HC. In the task-related phase, there was no such interaction (see S1.2 mood-congruency bias in happy–sad trials in the supplementary material), suggesting that the active cognitive focus on the face (up to the participant's responses) might indeed mask the mood-congruency bias. Overall, these results are consistent with the consensus from previous meta-analyses that MDD patients have decreased maintenance on positive faces [34, 35].

Second, we tested group differences in the comparisons of sad vs. other negative faces. Our *PTRA* analyses first provided eye-tracking evidence that MDD patients have more preferential attention to sad faces than other negative faces (e.g., fearful and angry), which indicated exclusively attentional maintenance on the mood-congruent faces but not generally negative faces. Moreover, this mood-congruency bias in MDD during the free-viewing phase was not affected by response times, dwell times, and fixation counts due to the absence of group differences in these indices (see “Behavioral results” and S1.4 Group differences in the dwell times and fixation counts in the supplementary material). Finally, there was no main effect of the group for preferential attention to negative faces in our studies, which argues against a generic negativity bias in MDD. This is in line with the cognitive model of depression [46, 47], which emphasizes that depressive self-referential schemas lead to selective attention to sad information. In addition, a recent study revealed gender differences in the mood-congruency bias [48]. We also examined these differences and did not observe any gender effects in mood-congruency bias in our eye-tracking analyses (see S1.3 Gender effects in mood-congruency bias in the supplementary material).

In line with the recent large-scale study mentioned above [18], there were no group differences in amygdala activity in response to emotional faces with geometric forms as the baseline either in free-viewing or task-guided processing (see Fig. 3). Nevertheless, we observed increased amygdala activity in response to emotional faces compared with geometric forms in both trial phases, reflecting robust amygdala involvement for processing emotional stimuli in contrast to neutral stimuli, consistent with multiple previous studies [2, 7–11]. Furthermore, we did observe increased amygdala activity in the free-viewing compared to the task-related phase. This might reflect a candidate role of the amygdala in spontaneous emotional processing, such as emotion regulation without explicit instruction. Schuyler et al. split the emotional processing into periods before and after the presentation of stimuli, and implicate that speed of amygdala change in prolonged periods (i.e., after ceasing presentation of stimuli), rather than initial periods, can differentiate individuals with mood dysregulation from healthy controls, as self-generated autonomous responses are accordingly associated with one’s own trait or clinical symptom when individuals no longer receive instruction [49]. This confirms the relationship between the amygdala and spontaneous affective processing, as well as supported by our results and the other work [50]. In addition, for the task-related phase, engaging in a task always involves executive control and decision-making, which may suppress spontaneous activity, as indicated by the frontoparietal network, default mode network, and attention network [51, 52].

When we compared the neural correlates of gaze patterns between the free-viewing and task-related phases via parametric modulations, our results did not reveal differences in the amygdala correlates of gaze patterns between the phases. However, we did observe differences in other brain regions: attentional preference was associated with clusters of activity in the (lateral) prefrontal cortex in the free-viewing phase, compared to greater activation in the primary visual cortex in the task-related phase (see Fig. 5b). This indicates that in our study, even though emotional processing dominated both processing phases manifesting in increased amygdala activity, the intrinsic natures of emotional processing of the two phases are rather different. Specifically, the task-related processing for identifying emotion appears more likely to be based on specific shapes (e.g., of eyes and mouth) among three faces in a trial, reflecting bottom-up processing. This processing probably resulted in a more primary visual cortical activity, which is in line with previous studies on stimulus-driven or feature-based visual searching [53, 54]. By contrast, processing in the free-viewing phase seems to involve a more spontaneous response dependent on an individual’s mood state or a self-referential scheme, possibly indicative of a more endogenous control compared to the task-related phase. Previous meta-analyses of the fMRI and PET measurements have implicated that self-referential processing, in particular, self-referential thinking is more likely to elicit activity in the prefrontal cortex, including the inferior and medial-frontal gyrus and the default mode network [55–58], which is consistent with our findings. Synthesizing the two contrasts, our findings demonstrating a shift in brain activity from the primary visual area to the prefrontal area implicated a transition from stimulus-driven perception in task-evoked processing to a high-order cognition in free-viewing processing.

However, our analyses did not reveal pronounced differential neural correlates between the groups. This is applied to the neural correlates of the task regressors (face > geometric form) and to the parametric modulations of *PTRA* in the task-related and the free-viewing phase. Only for the latter analysis, at a lower level of significance, when we collected our statistical maps at uncorrected *p* < 0.005 and performed a subsequent familywise error cluster correction, we noted differential neural correlates. The MDD patients had increased activity associated with *PTRA* in the middle and posterior cingulate, occipital, and calcarine gyri, and reduced activity in a cluster in the supplementary motor area (SMA, extending into the prefrontal cortex). This reflects reduced activity in the SMA to task-related face in MDD patients, suggesting that healthy controls recruit more activity in this cluster after providing the behavioral response, potentially reflecting on the target stimulus and response. By contrast, MDD patients revealed more posterior cingulate activity associated with the same posterior modulation in the same phase, suggestive of increased default mode network activity and self-referential processing. However, we refrain from over-interpreting this result as the significance threshold was rather low, which is in line with the notion that fMRI BOLD does not have as high precision as the eye-tracking measurement.

Moreover, there are a few limitations that warrant attention. First, a small number of trials were available to investigate the mood-congruency bias in gaze data, which might impact the reliability of the results. However, this low number of trials would be compensated by a relatively large sample size, which allowed our research to retain sufficient power. Second, the order of the processing phases was fixed, so the task-related phase always preceded the free-viewing phase in one trial. With the comparatively slow BOLD response modeled by a canonical hemodynamic response function, brain activity from an early phase may influence the response of its following phases [59]. Beyond this, brain activity in the free-viewing phase could, to some extent, reflect general rumination processes following mood induction by emotional faces in the task-related phase. A previous study indicates that rumination increases the self-referential processing in MDD [60]. To avoid the effects of delay and rumination, it would be helpful to adopt a block design that separates the processing states (free vs. task-evoked viewing) into different blocks.

## Conclusion

We observed altered emotional processing in MDD reflected in a mood-congruency attentional bias that was most pronounced in the free-viewing phase of an emotional face-matching task, although this altered processing was not associated with differences in amygdala activity. When comparing task-evoked to free-viewing processing, we observed a shift from relatively stronger activity in visual cortices to relatively stronger activity of (dorso-) lateral prefrontal cortices, potentially differentiating task-driven activity from more endogenous activity. Our results imply that the combination of eye-tracking and fMRI parameters may improve our understanding of the neural mechanism for disentangling emotional processes in MDD.

### Supplementary Information

Below is the link to the electronic supplementary material.

Supplementary file1 (DOCX 2466 KB)

## Data Availability

The data of this study, as a part of ongoing BeCOME project at the Max Planck Institute of Psychiatry (Munich, Germany), are available from the corresponding author upon reasonable request.

## References

[1] Insel T, Cuthbert B, Garvey M (2010). Research Domain Criteria (RDoC): Toward a new classification framework for research on mental disorders. Am J Psychiatry.

[2] Hariri AR, Tessitore A, Mattay VS (2002). The amygdala response to emotional stimuli: a comparison of faces and scenes. Neuroimage.

[3] Enhancing NeuroImaging Genetics through Meta-Analysis (ENIGMA) (1999) https://enigma.ini.usc.edu/ongoing/enigma-tbfmri/

[4] Dunedin Multidisciplinary Health & Development Research Unit (The Dunedin Study) (1999) https://dunedinstudy.otago.ac.nz/

[5] Human Connectome Project (2011) https://www.humanconnectome.org/

[6] UK Biobank (2012) https://www.ukbiobank.ac.uk/

[7] Elliott ML, Knodt AR, Ireland D (2020). What Is the test-retest reliability of common task-functional MRI measures? New empirical evidence and a meta-analysis. Psychol Sci.

[8] Matthews SC, Strigo IA, Simmons AN (2008). Decreased functional coupling of the amygdala and supragenual cingulate is related to increased depression in unmedicated individuals with current major depressive disorder. J Affect Disord.

[9] Norbury R, Selvaraj S, Taylor MJ (2010). Increased neural response to fear in patients recovered from depression: a 3T functional magnetic resonance imaging study. Psychol Med.

[10] Sauder CL (2013). Test-retest reliability of amygdala response to emotional faces. Early Hum Dev.

[11] Townsend JD, Eberhart NK, Bookheimer SY (2010). FMRI activation in the amygdala and the orbitofrontal cortex in unmedicated subjects with major depressive disorder. Psychiatry Res Neuroimaging.

[12] Carballedo A, Scheuerecker J, Meisenzahl E (2011). Functional connectivity of emotional processing in depression. J Affect Disord.

[13] Frodl T, Scheuerecker J, Albrecht J (2009). Neuronal correlates of emotional processing in patients with major depression. World Journal of Biological Psychiatry.

[14] Frodl T, Scheuerecker J, Schoepf V (2010). Different effects of mirtazapine and venlafaxine on brain activation: an open randomized controlled fMRI study. J Clin Psychiatry.

[15] Peluso MAM, Glahn DC, Matsuo K (2009). Amygdala hyperactivation in untreated depressed individuals. Psychiatry Res Neuroimaging.

[16] Scheuerecker J, Meisenzahl EM, Koutsouleris N (2010). Orbitofrontal volume reductions during emotion recognition in patients with major depression. J Psychiatry Neurosci.

[17] Zhong M, Wang X, Xiao J (2011). Amygdala hyperactivation and prefrontal hypoactivation in subjects with cognitive vulnerability to depression. Biol Psychol.

[18] Tamm S, Harmer CJ, Schiel J (2022). No association between amygdala responses to negative faces and depressive symptoms: cross-sectional data from 28,638 individuals in the UK Biobank Cohort. Am J Psychiatry.

[19] Fournier JC, Chase HW, Almeida J, Phillips ML (2014). Model specification and the reliability of fMRI results: implications for longitudinal neuroimaging studies in psychiatry. PLoS ONE.

[20] Gee DG, Mcewen SC, Forsyth JK (2015). Reliability of an fMRI paradigm for emotional processing in a multisite longitudinal study. Hum Brain Mapp.

[21] Plichta MM, Schwarz AJ, Grimm O (2012). Test-retest reliability of evoked BOLD signals from a cognitive-emotive fMRI test battery. Neuroimage.

[22] Sauder CL, Hajcak G, Angstadt M, Phan KL (2013). Test-retest reliability of amygdala response to emotional faces. Psychophysiology.

[23] Lipp I, Murphy K, Wise RG, Caseras X (2014). Understanding the contribution of neural and physiological signal variation to the low repeatability of emotion-induced BOLD responses. Neuroimage.

[24] van den Bulk BG, Cédric P, Koolschijn MP (2013). How stable is activation in the amygdala and prefrontal cortex in adolescence? A study of emotional face processing across three measurements. Dev Cogn Neurosci.

[25] Nord CL, Gray A, Charpentier CJ (2017). Unreliability of putative fMRI biomarkers during emotional face processing. Neuroimage.

[26] Boubela RN, Kalcher K, Huf W (2015). FMRI measurements of amygdala activation are confounded by stimulus correlated signal fluctuation in nearby veins draining distant brain regions. Sci Rep.

[27] Martinez-Zalacain I, Leon AL, del Cerro I (2019). Altered pupillary response during oddball detection in mild cognitive impairment and late-life major depression: neuroimaging correlates. Eur Neuropsychopharmacol.

[28] Schneider M, Elbau IG, Nantawisarakul T (2020). Pupil dilation during reward anticipation is correlated to depressive symptom load in patients with major depressive disorder. Brain Sci.

[29] Rusch KM (2021). Combining fMRI and eye-tracking for the study of social cognition. Neurosci Insights.

[30] Bradley BP, Mogg K, Falla SJ, Hamilton LR (1998). Attentional bias for threatening facial expressions in anxiety: manipulation of stimulus duration. Cogn Emot.

[31] Allard ES, Yaroslavsky I (2019). Attentional disengagement deficits predict brooding, but not reflection, over a one-year period. Front Psychol.

[32] Ellis AJ, Wells TT, Vanderlind WM, Beevers CG (2014). The role of controlled attention on recall in major depression. Cogn Emot.

[33] Grossheinrich N, Firk C, Schulte-Rüther M (2018). Looking while unhappy: a mood-congruent attention bias toward sad adult faces in children. Front Psychol.

[34] Armstrong T, Olatunji BO (2012). Eye tracking of attention in the affective disorders: a meta-analytic review and synthesis. Clin Psychol Rev.

[35] Suslow T, Hußlack A, Kersting A, Bodenschatz CM (2020). Attentional biases to emotional information in clinical depression: a systematic and meta-analytic review of eye tracking findings. J Affect Disord.

[36] Duque A, Vázquez C (2015). Double attention bias for positive and negative emotional faces in clinical depression: evidence from an eye-tracking study. J Behav Ther Exp Psychiatry.

[37] van Dillen LF, Heslenfeld DJ, Koole SL (2009). Tuning down the emotional brain: an fMRI study of the effects of cognitive load on the processing of affective images. Neuroimage.

[38] Brückl TM, Spoormaker VI, Sämann PG (2020). The biological classification of mental disorders (BeCOME) study: a protocol for an observational deep-phenotyping study for the identification of biological subtypes. BMC Psychiatry.

[39] Wittchen H, Beloch E (1997) DIA-X-Interview: Instruktionsmanual zur Durchführung von DIA-X-Interviews

[40] Ekman P, Friesen WV (1976). Pictures of facial affect.

[41] Mumford JA, Poline JB, Poldrack RA (2015). Orthogonalization of regressors in fMRI models. PLoS ONE.

[42] Rolls ET, Joliot M, Tzourio-Mazoyer N (2015). Implementation of a new parcellation of the orbitofrontal cortex in the automated anatomical labeling atlas. Neuroimage.

[43] Bistricky SL, Ingram RE, Atchley RA (2011). Facial affect processing and depression susceptibility: cognitive biases and cognitive neuroscience. Psychol Bull.

[44] Clasen PC, Wells TT, Ellis AJ, Beevers CG (2013). Attentional biases and the persistence of sad mood in major depressive disorder. J Abnorm Psychol.

[45] van Vleet T, Stark A, Merzenich MM et al (2020) Biases in processing of mood-congruent facial expressions in depression. Psychiatry Research 275:143–148. 10.1016/j.psychres.2019.02.076.Biases 10.1016/j.psychres.2019.02.076 PMC6504610 30908978

[46] Disner SG, Shumake JD, Beevers CG (2017). Self-referential schemas and attentional bias predict severity and naturalistic course of depression symptoms. Cogn Emot.

[47] Beck A (1987). Cognitive models of depression. J Cogn Psychother.

[48] Steephen JE, Kummetha S, Obbineni SC, Bapi RS (2021). Mood-congruent biases in facial emotion perception and their gender dependence. Int J Psychol.

[49] Schuyler BS, Kral TRA, Jacquart J (2014). Temporal dynamics of emotional responding: Amygdala recovery predicts emotional traits. Soc Cogn Affect Neurosci.

[50] Walter H, von Kalckreuth A, Schardt D (2009). The temporal dynamics of voluntary emotion regulation. PLoS ONE.

[51] Lynch LK, Lu KH, Wen H (2018). Task-evoked functional connectivity does not explain functional connectivity differences between rest and task conditions. Hum Brain Mapp.

[52] Gilson M, Deco G, Friston KJ (2018). Effective connectivity inferred from fMRI transition dynamics during movie viewing points to a balanced reconfiguration of cortical interactions. Neuroimage.

[53] Maunsell JHR, Treue S (2006). Feature-based attention in visual cortex. Trends Neurosci.

[54] Serences JT, Yantis S (2007). Spatially selective representations of voluntary and stimulus-driven attentional priority in human occipital, parietal, and frontal cortex. Cereb Cortex.

[55] Delaveau P, Jabourian M, Lemogne C (2011). Brain effects of antidepressants in major depression: a meta-analysis of emotional processing studies. J Affect Disord.

[56] Doucet GE, Janiri D, Howard R (2020). Transdiagnostic and disease-specific abnormalities in the default-mode network hubs in psychiatric disorders: a meta-analysis of resting-state functional imaging studies. Eur Psychiatry.

[57] Zhou HX, Chen X, Shen YQ (2020). Rumination and the default mode network: Meta-analysis of brain imaging studies and implications for depression. Neuroimage.

[58] Hu C, Di X, Eickhoff SB (2016). Distinct and common aspects of physical and psychological self-representation in the brain: a meta-analysis of self-bias in facial and self-referential judgements. Neurosci Biobehav Rev.

[59] Liao CH, Worsley KJ, Poline JB (2002). Estimating the delay of the fMRI response. Neuroimage.

[60] Nejad AB, Fossati P, Lemogne C (2013). Self-referential processing, rumination, and cortical midline structures in major depression. Front Hum Neurosci.

