# Supplementary material for: Free-viewing gaze patterns reveal a mood-congruency bias in MDD during an affective fMRI/eye-tracking task
Source: Eur Arch Psychiatry Clin Neurosci. 2023 Apr 23;274(3):559–71. doi: 10.1007/s00406-023-01608-8 (PMC10995059; doi:10.1007/s00406-023-01608-8)
Supplement: Supplementary file 1 — Supplementary file1 (DOCX 2466 KB) [file 406_2023_1608_MOESM1_ESM.docx]

**Supplementary Material**

Free-viewing gaze pattern reveals a mood-congruency bias in MDD during an emotional fMRI/eye-tracking task

Rui Sun^1,2^, Julia Fietz^1,3^, Mira Erhart,^1,3^, Dorothee Poehlchen^1,3^, Lara Henco^1^, Tanja M. Brückl^1^，BeCOME study team^1‡^, Michael Czisch^1^, Philipp G. Saemann^1^, Victor I. Spoormaker^1*^

Affiliations: ^1^ Department of Translational Research in Psychiatry, Max Planck Institute of Psychiatry, Munich, Germany; ^2^ Department of Behavioral and Psychological Science, Zhejiang University, Hangzhou, China; ^3^International Max Planck Research School for Translational Psychiatry (IMPRS-TP), Max Planck Institute of Psychiatry, Munich, Germany

^*^Corresponding Author: Victor I. Spoormaker

E-mail address: spoormaker@psych.mpg.de

^‡^ BeCOME Working Group: Elisabeth B. Binder, Angelika Erhardt, Susanne Lucae, Norma C. Grandi, Tamara Namendorf, Immanuel Elbau, Laura Leuchs, Anna Katharine Brem, Leonhard Schilbach, Sanja Ilić-Ćoćić, Julius Ziebula, Iven-Alex von Mücke-Heim, Yeho Kim and Julius Pape.

**Outlines**

**Part 1: Additional Analysis**

S1.1 Group differences in accuracy rates

S1.2 Mood-congruency bias in happy-sad trials

S1.3 Gender effects in mood-congruency bias

S1.4 Group differences in the dwell times and fixation counts

**Part 2: Figures**

**Fig.S1** Flowchart of exclusion of participants for the measurements

**Fig. S2** Interaction between group and emotion of target faces in the task-related phase (a) and the free-viewing phase (b)

**Fig. S3** Neural correlates of *PTRA* for emotional faces between the groups (MDD vs. HC) in the task-related phase (a) and the free-viewing phase (b)

**Fig. S4** Neural correlates of task-related attentional gaze patterns during the task-related phase in MDD (a) and HC (b)

**Fig. S5** Neural correlates of task-related attentional gaze patterns during the free-viewing phase in MDD (a) and HC (b)

**Part 3: Tables**

Table S1 Demographic information, RTs, and accuracy rates in the behavioral analyses

Table S2 Demographic information and *PTRA* values in the eye-tracking analyses

Table S3 Demographic information in the fMRI analyses

Table S4 Brain activity in response to emotional faces (vs. geometric forms) in the task-related phase

Table S5 Brain activity in response to emotional faces (vs. geometric forms) in the free-viewing phase

Table S6 Brain activity in response to emotional faces in the free-viewing phase vs. in the task-related phase

Table S7 neural correlates of *PTRA* (only for emotional faces) in the free-viewing phase vs. in the task-related phase

Table S8 Neural correlates of *PTRA* between the groups (MDD vs. HC) in the task-related phase

Table S9 Neural correlates of *PTRA* between the groups (MDD vs. HC) in the free-viewing phase

Table S10 Neural correlates of *PTRA* (only for emotional faces) for MDD in the task-related phase

Table S11 Neural correlates of *PTRA* (only for emotional faces) for HC in the task-related phase

Table S12 Neural correlates of *PTRA* (only for emotional faces) for MDD in the free-viewing phase

Table S13 Neural correlates of *PTRA* (only for emotional faces) for HC in the free-viewing phase

**Part 1: Additional Analysis**

**S1.1 Group differences in accuracy rates**

To test for possible differences in accuracy for different stimulus types, we first conducted a two-factorial ANOVA (factor *group*, two levels: MDD vs. HC; factor *stimulus*, two levels: emotional face vs. geometric form). The results showed that there was a significant main effect of stimulus (*F* _(1,188)_ = 61.95, *p* < .001, *η*^2^_p_ = .27), indicating participants had higher accuracy in shape recognition of geometric forms than facial emotion recognition (*t*_(189)_ = 7.87, *p* < .001, Cohen’s d = .84). However, the main effect of group and interaction between group and stimulus were not significant (*F*s < .84, *p*s > .36), with accuracy rates for the two groups in both conditions over 90%.

Then, we conducted a two-factorial ANOVA (factor *group*, two levels: MDD vs. HC; factor *emotional pair*, two levels: positive-negative vs. negative-negative) to test the group differences for different emotional pairs. The results showed that there was a significant main effect of emotional pair (*F* _(1,188)_ = 94.72, *p* < .001, *η*^2^_p_ = .13), but again the main effect of group and the interaction between group and emotional pair were not significant (*F*s < .41, *p*s > .52).

Last, we conducted a two-factorial ANOVA (factor *group*, two levels: MDD vs. HC; factor *specific-valenced pair*, two levels: happy-sad vs. happy-angry-fearful) for testing the mood-congruency bias from the perspective of accuracy. The results showed that there were no significant main effects of group and specific-valenced pair (*F*s < 0.36, *p*s > .55,), but there was an interaction between group and specific-valenced pair (*F* _(1,188)_ = 6.59, *p* = .01, *η*^2^_p_ = .04). The post hoc analysis revealed that healthy participants had marginally higher recognition accuracy for happy-sad faces than happy-angry-fearful faces (*t*_(124)_ = 2.68, *p* = .049), but there was no such significant comparison in MDD, with all accuracy rates above 0.98. Besides, the post hoc analysis did not show any group differences in each type of specific-valenced pair. As this was in the task-related phase, we added this analysis as an effect of potential interest but found little relation to the mood congruency bias in the free-viewing phase.

In summary, the MDD and the HC group had similar accuracy in emotion and shape recognition, with accuracy rates between 90-100%.

**S1.2 Mood-congruency bias in happy-sad trials**

To test whether a mood-congruency bias could be reflected by variance in preferential attention to sad faces or happy faces between MDD and HC. We conducted a two-factorial ANOVA (factor *group*, two levels: MDD vs. HC; factor *target face*, two levels: happy vs. sad) for both phases. Results showed that in the free-viewing phase, the interaction between group and target face is significant (*F*_(1, 170)_  = 4.37, *p* = .04, *η^2^*_p_ > .02). Post hoc test showed that MDD had less *PTRA* for happy faces than HC, indicating decreased maintenance on happy faces for MDD than HC (*t* = 2.63, *p* = .05, Cohen’s d = .40), supportive of a mood congruency in MDD. However, in the task-related phase, there is no significant interaction or main effect (*F*_(1, 170)_  < 2.34, *p* > .13, see Fig. S2).

**S1.3 Gender effects in mood-congruency bias**

To test for gender differences in the mood-congruency bias, we conducted a three-factorial ANOVA (factor group, two levels: MDD vs. HC; factor gender, two levels: female vs. male; factor specific-valenced pair, two levels: happy-sad vs. happy-angry-fearful) in each phase (the task-related phase or the free-viewing phase), with the proportion of target-related attention (*PTRA*) as the dependent variable. The results showed that in the task-related phase, there was no significant main effect of gender, no significant interaction between gender and group, no significant interaction between gender and specific-valenced pair, and no significant interaction among all three factors (i.e., group, gender, and specific-valenced pair, Fs < 2.29, ps > 0.13). In the free-viewing phase, there was a trend for a significant interaction among all three factors (F_(1, 166)_ = 3.31, p = 0.07), although the main effect of gender and other interactions with gender weren’t significant (Fs < 2.03, ps > 0.16). For the post hoc analysis of the marginally significant interaction, there were no significant differences in any comparisons (ts < 2.90, ps > 0.11). Therefore, in the task-related or free-viewing phase of emotional processing, we didn’t observe gender differences between and within the MDD and the HC group for the *PTRA* values in happy-negative trials.

Then we also tested the gender effect in the happy-sad trials with the same condition as in the supplementary material. This analysis focused on testing the mood-congruency attentional bias between positive (happy) faces and negative (sad) faces, which was different from the analysis above (examining between mood-congruent negative (sad) faces and other negative (fearful and angry) faces). We conducted a three-factorial ANOVA (factor *group*, two levels: MDD vs. HC; factor *gender*, two levels: female vs. male; factor *target face*, two levels: happy vs. sad) in each phase, also with *PTRA* values as the dependent variable. The results showed that in the task-related phase, the main effect of gender, the interaction between gender and target face, and the interaction among three factors (i.e., group, gender, and target face) were not significant (*F*s < .42, *p*s > 0.52), but the interaction between gender and group showed a trend for significance (*F*_(1, 166)_ = 3.46, *p* = 0.06, *η*^2^_p_ =.002). The post hoc analysis showed that for female participants, the HC group had marginally higher *PTRA* values than the MDD group (*t*_(168)_ = 2.48, *p* = .08), indicating that healthy female individuals have more attention to target-related faces than MDD female patients in the task-related phase. However, there were no significant differences in other comparisons of the post hoc analysis (*t*s < 2.08, *p*s > .24). In the free-viewing phase, there was no main effect of gender, no interaction between gender and target face, and no interaction between gender and group (*F*s< 1.71, *p*s > 0.19), but the interaction among the three factors was marginally significant (*F*_(1, 166)_ = 3.06, *p* = 0.08, *η*^2^_p_ =.002). For the post hoc analysis, there were no significant differences in any comparisons (*t*s < 2.75, *p*s > .18).

In summary, we didn’t observe any significant interactions among three factors (i.e., group, gender, and specific-valenced pair (or target face)), indicating mood congruency bias cannot be distinguished by gender either for MDD or non-clinical populations in this study. This demonstrated that both female and male patients with MDD had a more attentional bias to mood-congruent faces during the free-viewing, as compared with their counterparts.

**S1.4 Group differences in the dwell times and fixation counts**

Differences in dwell times and fixation counts between groups may influence mood-congruency attentional bias. To test for possible group differences, we conducted four two-factorial ANOVAs (factor *group*, two levels: MDD vs. HC; factor *specific-valenced pair*, two levels: happy-sad vs. happy-angry-fearful) respectively for dwell times and fixation counts in each phase (i.e., the task-related phase or the free-viewing phase).

Dwell times:

The two-factorial ANOVA showed a significant main effect of group for dwell times in the task-related phase (*F*_(1,168)_ = 4.06, *p* = .045), indicating that patients with MDD had shorter dwell times on stimuli than healthy controls. However, there was no significant main effect of group in the free-viewing phase (*F* = .05, *p* = .83). In addition, there were no interactions between the group and specific-valenced pair in each phase (*F*s *<* 1.95, *p*s > .16).

To further test the effect of phase, we conducted a three-factorial ANOVA (factor *phase*, two levels: task-related vs. free-viewing; factor *group*, two levels: MDD vs. HC; factor *specific-valenced pair*, two levels: happy-sad vs. happy-angry-fearful). Results showed that the main effect of phase was significant (*F*_(1,168)_ = 632.15, *p* < .001, *η*^2^_p_ = .74), indicating that participants had shorter dwell times in the task-related phase than the free-viewing phase. The main effect of specific-valenced pair and interaction between phase and specific-valenced pair were significant (*F*s > 9.38, *p*s < .002, *η*^2^_p_ > .04). However, the main effect of group and interactions with group were not significant (*F*s *<* 2.26, *p*s > .13).

Fixation counts:

The two-factorial ANOVA showed there was no significant main effect of group either in the task-related phase (*F* = .06, *p* = .80) or in the free-viewing phase (*F*_(1,168)_ = 1.03, *p* = .31). In addition, there were no interactions between the group and specific-valenced pair in each phase (*Fs >* .93, *p*s > .34).

Similarly, to further test the effect of phase, we conducted a three-factorial ANOVA (factor *phase*, two levels: task-related vs. free-viewing; factor *group*, two levels: MDD vs. HC; factor *specific-valenced pair*, two levels: happy-sad vs. happy-angry-fearful) for fixation counts. Results also showed that the main effect of phase was significant (*F*_(1,168)_ = 347.52, *p* < .001, *η*^2^_p_ = .61), indicating that participants had fewer fixations in the task-related phase than the free-viewing phase. The main effect of specific-valenced pair and interaction between phase and specific-valenced pair were significant (*F*s > 11.91, *p*s < .001, *η*^2^_p_ > .05). However, the main effect of group and interactions with group were not significant (*F*s *<* 1.32, *p*s > .25).

In summary, the MDD and the healthy group had similar dwell times and fixation counts in the free-viewing phase, which was not affected by the stimulus types. Therefore, mood-congruency attention bias between MDD and HC cannot be accounted for by dwell times or fixation counts on stimuli during the free-viewing phase. However, we observed that healthy participants had more accuracy for happy-sad faces than happy-angry-fearful faces, while there was no such significant comparison in MDD. The accuracy level was dominated mainly by task-related attention before responses, whereas the mood-congruency bias in our eye-tracking analyses was only observed in the free-viewing phase (after responses). Therefore, significant results of accuracy cannot provide further explanation for the mood-congruency attention bias in MDD in our study. In addition, the two groups have similar accuracy for stimuli, demonstrating that the effect of task difficulty did not affect the results of mood-congruency attention bias in MDD.

**Part 2: Figures**


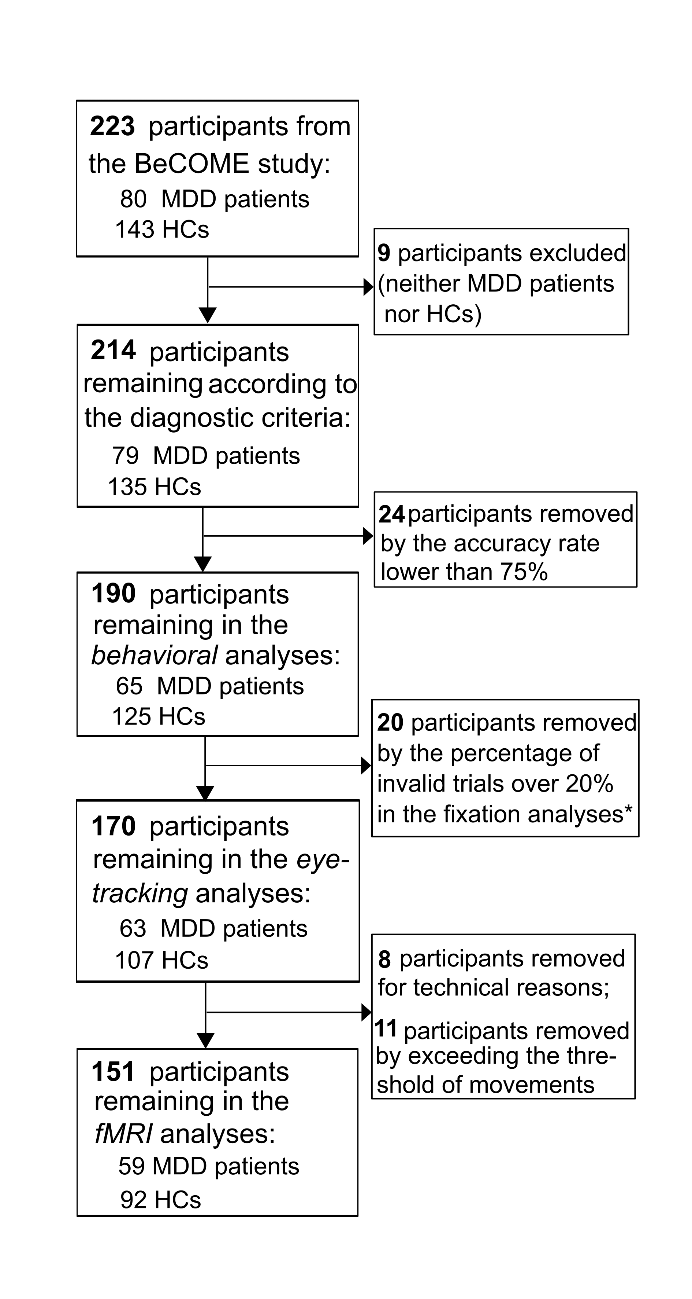


**Fig. S1** Flowchart of exclusion of participants for the measurements. The BeCOME study refers to the Biological Classification of Mental Disorders study. MDD and HC refer to major depressive disorder patients and healthy controls, respectively. * the fixation analyses were used to exclude the invalid trials in the eye-tracking analyses; a trial was excluded if more than 25% of fixations were located outside the regions of interest (ROI)


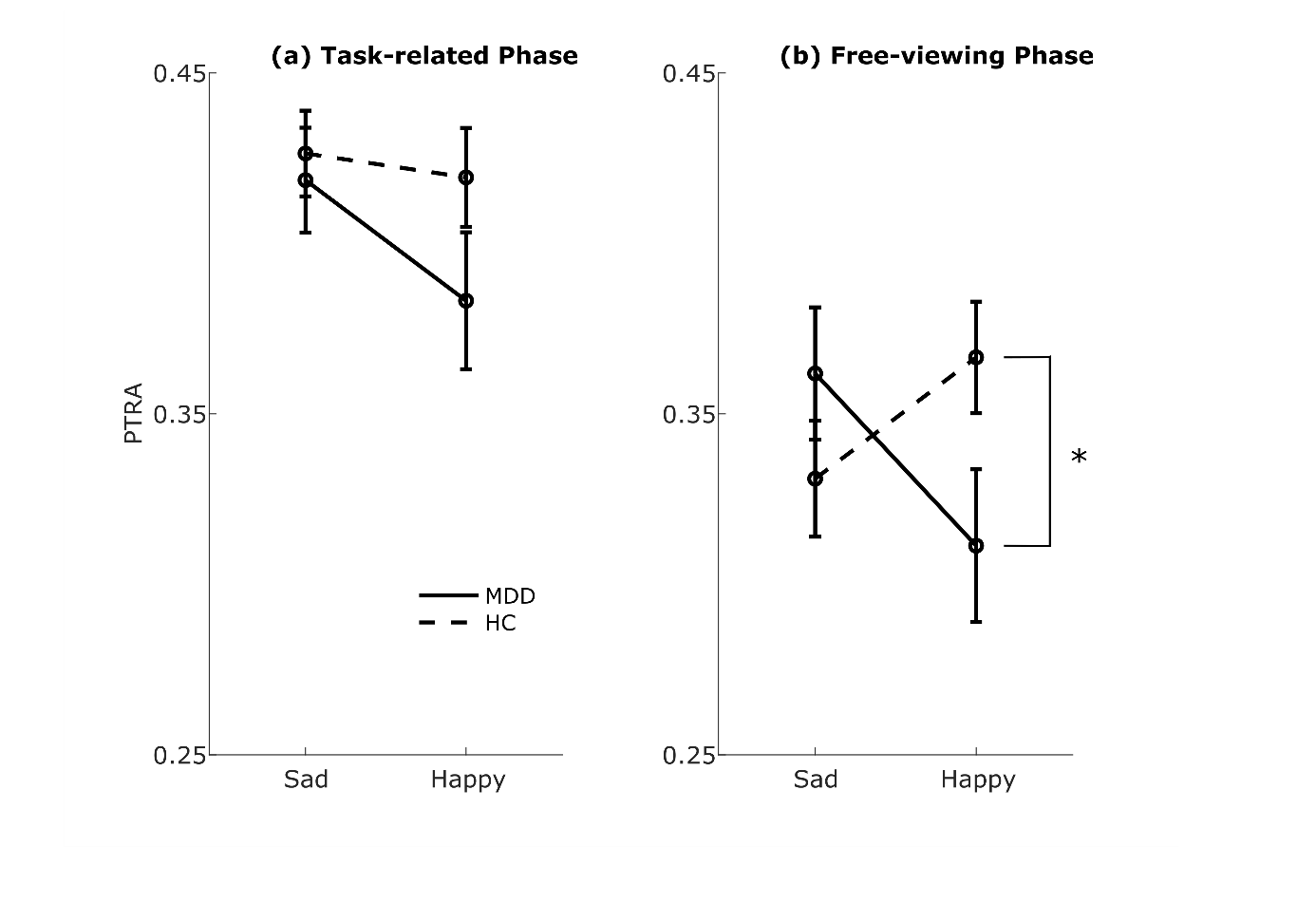


**Fig. S2** Interaction between group and target face in the task-related phase (a) and the free-viewing phase (b). MDD patients had lower *PTRA* for happy faces than HCs in the free-viewing phase, indicating that MDD had decreased attentional maintenance on happy faces, supportive of a mood congruency bias in MDD. However, there was no significant interaction in the task-related phase. *PTRA*: the proportion of target-related attention; MDD: major depression disorder; HC: healthy control


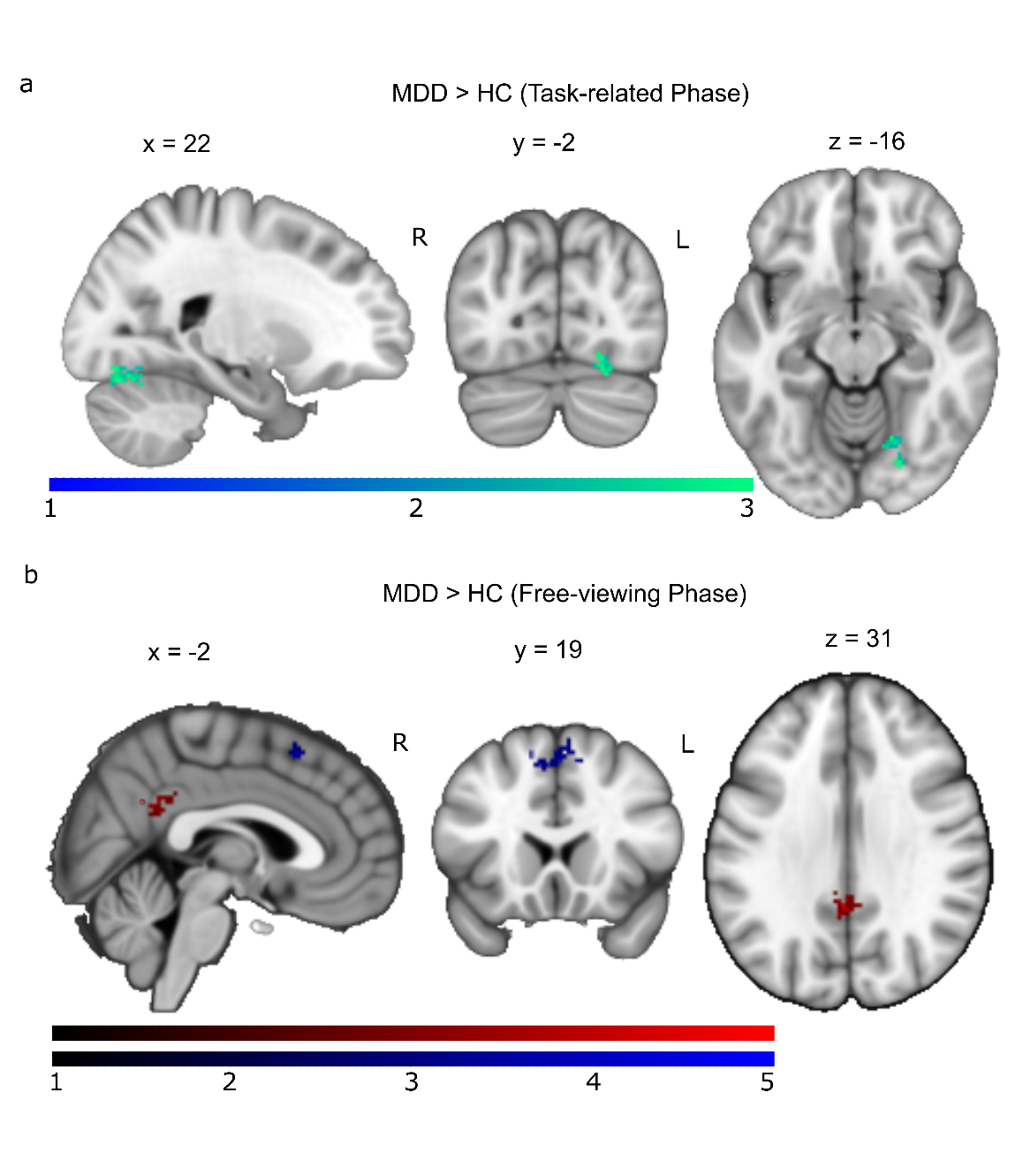


**Fig. S3** Neural correlates of *PTRA* for emotional faces between the groups (MDD vs. HC) in the target-related phase (a) and the free-viewing phase (b). Hot (cold) colors refer to more (less) neural correlates for MDD patients compared to the HCs. The background template is a mean T1-weighted image with DARTEL spatial normalization. L = left, R = right. Color bars indicate T values. *p*_cluster.FWE_ < .05, with statistical maps collected at uncorrected *p* < .005 and *k* > 50


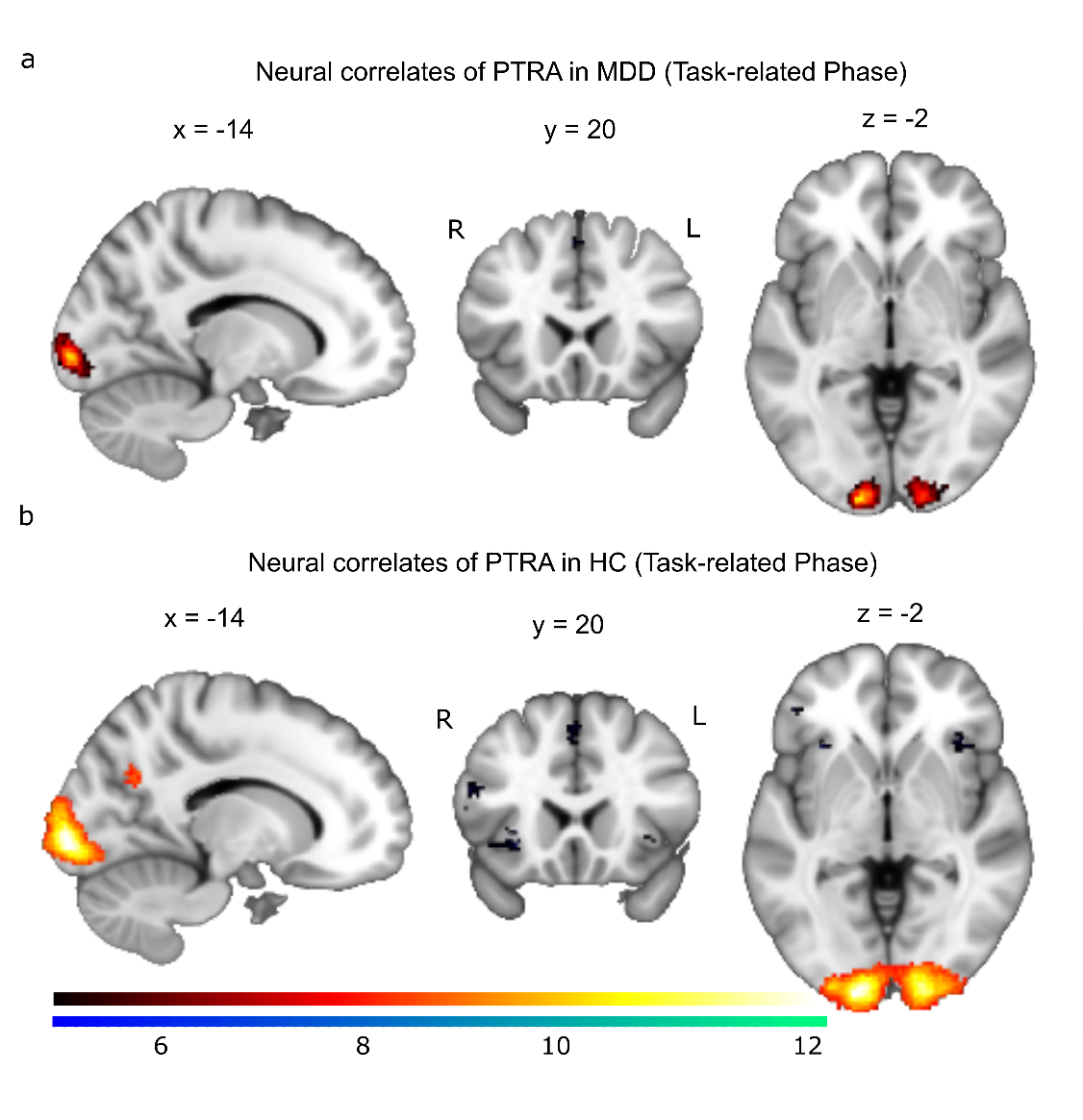


**Fig. S4** Neural correlates of task-related attentional gaze patterns during the task-related phase in MDD (a) and HC (b). Hot (cold) colors refer to positive (negative) neural correlates of gaze patterns in emotional face processing. The background template is a mean T1-weighted image with DARTEL spatial normalization. L = left, R = right. Color bars indicate T values. *p*_FWE_ < .05, *k* > 30


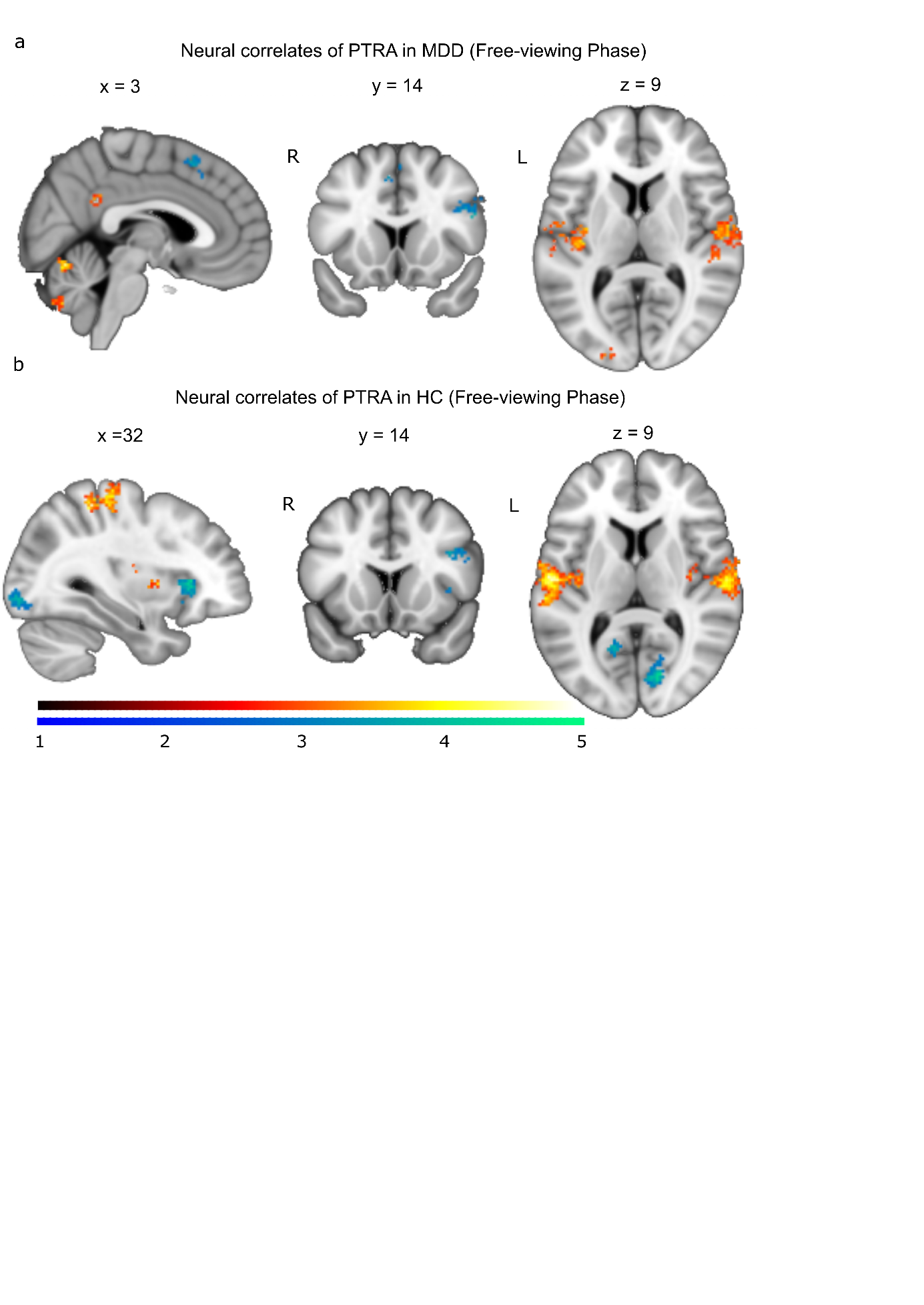


**Fig. S5** Neural correlates of task-related attentional gaze patterns during the free-viewing phase in MDD (a) and HC (b). Hot (cold) colors refer to positive (negative) neural correlates of gaze patterns in emotional face processing. The background template is a mean T1-weighted image with DARTEL spatial normalization. L = left, R = right. Color bars indicate T values. *p*_cluster.FWE_ < .05, with statistical maps collected at uncorrected *p* < .005 and *k* > 50

**Part 3: Tables**

**Table S1 Demographic information, RTs, and accuracy rates in the behavioral analyses**

|  | | MDD | |  | HC | |
| --- | --- | --- | --- | --- | --- | --- |
| n | | 65 | |  | 125 | |
| % Female | | 40.0 | |  | 30.4 | |
|  | | Male | Female |  | Male | Female |
| Age (years, *M*±*SD*) | | 35.7±14.4 | 34.5±12.6 |  | 31.6±9.7 | 35.4±13.6 |
|  | | RTs (ms) | Accuracy Rates |  | RTs (ms) | Accuracy Rates |
| Condition  (*M*±*SD*) | Emotional Faces | 2372.2±467.2 | .93±.08 |  | 2334.9±500.7 | .93±.07 |
|  | Geometric Forms | 1118.8±290.7 | .97±.04 |  | 1097.2±250.8 | .99±.03 |
|  | Positive-Negative | 2059.5±428.6 | .96±.07 |  | 2012.0±448.6 | .97±.07 |
|  | Negative-Negative | 2684.9±570.8 | .90±.11 |  | 2657.9±604.0 | .90±.10 |
|  | Happy-Sad* | 1729.5±439.5 | .98±.09 |  | 1627.0±453.8 | 1.00±.05 |
|  | Happy-Angry-Fearful* | 1899.3±461.5 | 1.00±.03 |  | 1859.8±497.5 | .98±.08 |

*Note:* MDD and HC refer to major depressive disorder patients and healthy controls; RTs refer to response times; * refers to the face pairs with a happy target (top face); *M* and *SD* represent the mean score and standard deviation; ms represent milliseconds.

**Table S2 Demographic information and *PTRA* values in the eye-tracking analyses**

|  | | MDD | |  | HC | |
| --- | --- | --- | --- | --- | --- | --- |
| n | | 63 | |  | 107 | |
| % Female | | 39.7 | |  | 31.8 | |
|  | | Male | Female |  | Male | Female |
| Age (years, *M*±*SD*) | | 35.8±14.0 | 33.5±11.9 |  | 32.5±10.3 | 34.8±13.2 |
|  | | TRP | FVP |  | TRP | FVP |
| Condition  (*PTRA*, *M*±*SD*) | Emotional Faces | .41±.12 | .32±.11 |  | .42±.13 | .34±.21 |
|  | Geometric Forms | .73±.29 | .30±.15 |  | .76±.29 | .34±.17 |
|  | Happy-Sad* | .40±.23 | .31±.26 |  | .44±.20 | .36±.22 |
|  | Happy-Fearful* | .41±.20 | .40±.21 |  | .41±.20 | .36±.22 |
|  | Happy-Angry* | .46±.18 | .40±.21 |  | .43±.19 | .38±.23 |
|  | Happy-Angry-Fearful* | .44±.16 | .40±.17 |  | .42±.17 | .38±.18 |

*Note:* *PTRA* refers to the proportion of target-related attention; MDD and HC refer to major depressive disorder patients and healthy controls; TRP and FVP represent the task-related phase and the free-viewing phase, respectively; * refers to the face pairs with a happy target (top face); *M* and *SD* refer to the mean score and standard deviation.

**Table S3 Demographic information in the fMRI analyses**

|  | MDD | |  | HC | |
| --- | --- | --- | --- | --- | --- |
| n | 59 | |  | 92 | |
| % Female | 43.4 | |  | 35.9 | |
|  | Male | Female |  | Male | Female |
| Age (years, *M*±*SD*) | 34.5±14.1 | 33.3±12.1 |  | 33.8±11.2 | 35.1±12.9 |

*Note:* MDD and HC represent major depressive disorder patients and healthy controls; *M* and *SD* represent the mean score and standard deviation.

**Table S4 Brain activity in response to emotional faces (vs. geometric forms) in the task-related phase**

| Emotional faces > Geometric forms | | | | | |
| --- | --- | --- | --- | --- | --- |
|  | x | y | z | *p*_voxel.FWE_ | *k* |
| Left inferior occipital gyrus (IOG) | -26 | -96 | -8 | < 10^-16^ | 8605 |
| Right inferior frontal gyrus (OpIFG) | 42 | 16 | 26 | < 10^-16^ | 6544 |
| left inferior frontal gyrus (OpIFG) | -54 | 22 | 18 | < 10^-16^ | 4397 |
| Left superior temporal gyrus (STG) | -54 | -46 | 8 | < 10^-16^ | 2192 |
| Left amygdala | -18 | -6 | -16 | < 10^-16^ | 225 |
| Left superior frontal gyrus medial segment (MSFG) | -2 | 32 | 48 | < 10^-16^ | 1327 |
| Right precuneus | 4 | -64 | 32 | < 10^-16^ | 609 |
| Right middle frontal gyrus (MFG) | 36 | 62 | 10 | 3.34×10^-11^ | 33 |
| Emotional faces < Geometric forms | | | | | |
|  | x | y | z | *p*_voxel.FWE_ | *k* |
| Right superior occipital gyrus (SOG) | 24 | -86 | 18 | < 10^-16^ | 5980 |
| Left supramarginal gyrus (SMG) | -58 | -28 | 38 | < 10^-16^ | 12036 |
| Left supplementary motor cortex (SMC) | -4 | -10 | 50 | < 10^-16^ | 1785 |
| Right anterior insula (AIns) | 42 | 4 | 2 | < 10^-16^ | 925 |
| Right cerebellum exterior | 12 | -70 | -48 | < 10^-16^ | 1520 |
| Right inferior temporal gyrus (ITG) | 54 | -58 | -10 | < 10^-16^ | 296 |
| Left cerebellum exterior | -14 | -66 | -50 | < 10^-16^ | 184 |
| Right precentral gyrus (PrG) | 30 | -14 | 62 | < 10^-16^ | 182 |
| Left cerebellum exterior | -24 | -58 | -26 | < 10^-16^ | 192 |
| Left fusiform gyrus (FuG) | -28 | -52 | -8 | < 10^-16^ | 151 |
| Right lingual gyrus (LiG) | 16 | -74 | -6 | < 10^-16^ | 75 |

**Table S5 Brain activity in response to emotional faces (vs. geometric forms) in the free-viewing phase**

| Emotional faces > Geometric forms | | | | | |
| --- | --- | --- | --- | --- | --- |
|  | x | y | z | *p*_voxel.FWE_ | *k* |
| Right inferior occipital gyrus (IOG) | 30 | -94 | -4 | < 10^-16^ | 8226 |
| Right inferior frontal gyrus (OpIFG) | 42 | 24 | 22 | < 10^-16^ | 2355 |
| Right amygdala | 24 | -4 | -16 | < 10^-16^ | 297 |
| Left amygdala | -28 | -4 | -20 | < 10^-16^ | 259 |
| Left inferior frontal gyrus (OpIFG) | -56 | 20 | 24 | < 10^-16^ | 2000 |
| Left middle temporal gyrus (MTG) | -56 | -42 | -42 | < 10^-16^ | 1022 |
| Right middle temporal gyrus (MTG) | 60 | -46 | -46 | < 10^-16^ | 861 |
| Right superior temporal gyrus (STG) | 54 | -8 | -14 | < 10^-16^ | 100 |
| Right medial frontal cortex (MFC) | 4 | 60 | -14 | < 10^-16^ | 178 |
| Left superior frontal gyrus medial segment (MSFG) | -4 | 54 | 40 | < 10^-16^ | 520 |
| Right temporal pole (TMP) | 54 | 10 | -24 | 8.35×10^-13^ | 40 |
| Right cerebellum | 24 | -80 | -36 | 2.91×10^-11^ | 32 |
| Emotional faces < Geometric forms | | | | | |
|  | x | y | z | *p*_voxel.FWE_ | *k* |
| Left superior occipital gyrus (SOG) | -22 | -92 | 20 | < 10^-16^ | 8990 |
| Left middle cingulate gyrus (MCgG) | -2 | 6 | 40 | < 10^-16^ | 715 |
| Right fusiform gyrus (FuG) | 24 | -52 | -14 | < 10^-16^ | 170 |
| Right cerebellum exterior | 16 | -66 | -54 | < 10^-16^ | 278 |
| Right supramarginal gyrus | 48 | -36 | 54 | < 10^-16^ | 402 |
| Left cerebellum exterior | -16 | -68 | -56 | 1.02×10^-13^ | 45 |
| Right cerebellum exterior | 36 | -46 | -46 | < 10^-16^ | 224 |
| Left percental gyrus (PrG) | -30 | -14 | 64 | < 10^-16^ | 146 |
| Right lingual gyrus (LiG) | 14 | -76 | -6 | < 10^-16^ | 96 |
| Left planum temporal (PT) | -48 | -40 | 18 | 1.05×10^-13^ | 45 |
| Right planum polare (PP) | 58 | 0 | -2 | < 10^-16^ | 258 |
| Left fusiform gyrus (FuG) | -26 | -48 | -10 | < 10^-16^ | 84 |
| Left cerebellum exterior | 40 | -54 | -46 | < 10^-16^ | 80 |

**Table S6 Brain activity in response to emotional faces in the free-viewing phase vs. in the task-related phase**

| Free-viewing phase > Task-related phase | | | | | |
| --- | --- | --- | --- | --- | --- |
|  | x | y | z | *p*_voxel.FWE_ | *k* |
| Right central operculum (CO) | 40 | -16 | 18 | < 10^-16^ | 16692 |
| Left angular gyrus (AnG) | -44 | -66 | 26 | < 10^-16^ | 11412 |
| Left cuneus | -6 | -66 | 26 | < 10^-16^ | 1266 |
| Left hippocampus | -26 | -20 | -20 | < 10^-16^ | 960 |
| Left middle frontal gyrus (MFG) | -36 | 16 | 52 | < 10^-16^ | 1240 |
| Left posterior cingulate gyrus (PCgG) | -4 | -38 | 36 | < 10^-16^ | 544 |
| Right middle frontal gyrus (MFG) | 52 | 44 | 14 | < 10^-16^ | 566 |
| Right middle frontal gyrus (MFG) | 26 | 22 | 50 | < 10^-16^ | 625 |
| Left cerebellum exterior | -18 | -46 | -58 | < 10^-16^ | 147 |
| Left middle frontal gyrus (MFG) | -44 | 54 | -2 | < 10^-16^ | 110 |
| Right central operculum (CO) | 36 | 4 | 12 | 3.60×10^-11^ | 35 |
| Left posterior orbital gyrus (POrG) | -32 | 34 | 12 | < 10^-16^ | 83 |
| Right medial orbital gyrus (MOrG) | 22 | 32 | -18 | 1.44×10^-15^ | 62 |
| Left superior frontal gyrus (SFG) | -8 | 64 | 12 | < 10^-16^ | 283 |
| Left temporal pole (TMP) | -46 | 8 | -34 | < 10^-16^ | 93 |
| Right middle temporal gyrus (MTG) | 54 | 0 | -38 | 1.28×10^-10^ | 32 |
| Left medial frontal cortex (MFC) | -6 | 52 | -10 | 5.47×10^-11^ | 34 |
| Free-viewing phase < Task-related phase | | | | | |
|  | x | y | z | *p*_voxel.FWE_ | *k* |
| Left anterior insula (AIns) | -30 | 22 | 0 | < 10^-16^ | 751 |
| Right anterior insula (AIns) | 32 | 26 | 0 | < 10^-16^ | 870 |
| Right precentral gyrus (PrG) | 42 | 4 | 30 | < 10^-16^ | 520 |
| Left cerebellum exterior | -34 | -54 | -32 | < 10^-16^ | 101 |
| Left cerebellum exterior | 34 | -54 | -30 | < 10^-16^ | 261 |
| Right lateral ventricle | 28 | -48 | 12 | < 10^-16^ | 194 |
| Left pallidum | -16 | 6 | 0 | < 10^-16^ | 89 |
| Left occipital fusiform gyrus (OFuG) | -26 | -78 | -8 | 3.33×10^-16^ | 66 |
| Left calcarine cortex (Calc) | -16 | -74 | 8 | < 10^-16^ | 107 |
| Left lateral ventricle | -26 | -50 | 14 | < 10^-16^ | 104 |
| Right middle frontal gyrus (MFG) | -30 | 38 | 30 | < 10^-16^ | 82 |

**Table S7 Neural correlates of *PTRA* (only for emotional faces) in the free-viewing phase vs. in the task-related phase**

| Free-viewing phase > Task-related phase | | | | | |
| --- | --- | --- | --- | --- | --- |
|  | x | y | z | *p*_voxel.FWE_ | *k* |
| Left inferior frontal gyrus (OpIFG) | -52 | 16 | 24 | < 10^-16^ | 350 |
| Left triangular part of the inferior frontal gyrus (TrIFG) | -44 | 40 | 0 | < 10^-16^ | 210 |
| Left supplementary motor cortex (SMC) | -6 | 24 | 44 | < 10^-16^ | 310 |
| Right cerebellum exterior | 10 | -76 | -26 | < 10^-16^ | 188 |
| Free-viewing phase < Task-related phase | | | | | |
|  | x | y | z | p_voxel.FWE_ | k |
| Right lingual gyrus (LiG) | 14 | -90 | -6 | < 10^-16^ | 2894 |
| Left precuneus (PCu) | -12 | -66 | 28 | < 10^-16^ | 60 |

**Table S8 Neural correlates of *PTRA* between the groups (MDD vs. HC) in the task-related phase**

| MDD < HC | | | | | |
| --- | --- | --- | --- | --- | --- |
|  | x | y | z | *p*_cluster.FWE *_ | *k* |
| Right cerebellum exterior | 22 | -76 | 12 | 4.60×10^-2^ | 91 |

*Note:* * Statistical maps were collected at a threshold of uncorrected *p* < 0.005, *k* > 50.

**Table S9 Neural correlates of *PTRA* between the groups (MDD vs. HC) in the free-viewing phase**

| MDD > HC | | | | | |
| --- | --- | --- | --- | --- | --- |
|  | x | y | z | *p*_cluster.FWE *_ | *k* |
| Left posterior cingulate gyrus (PoCgG) | -2 | -46 | 32 | 1.00×10^-3^ | 100 |
| Left inferior occipital gyrus (IOG) | -18 | -92 | -6 | 3.84×10^-5^ | 142 |
| MDD < HC | | | | | |
|  | x | y | z | *p*_cluster.FWE *_ | *k* |
| Right supplementary motor cortex (SMC) | 2 | 20 | 52 | 3.30×10^-2^ | 63 |

*Note:* * Statistical maps were collected at a threshold of uncorrected *p* < 0.005, *k* > 50.

**Table S10 Neural correlates of *PTRA* (only for emotional faces) for MDD in the task-related phase**

| Positive activity | | | | | |
| --- | --- | --- | --- | --- | --- |
|  | x | y | z | *p*_voxel.FWE_ | *k* |
| Middle occipital gyrus (MOG) | -14 | -98 | -2 | < 10^-16^ | 661 |
| Right calcarine (RC) | 16 | -98 | 0 | < 10^-16^ | 676 |
| Negative activity | | | | | |
|  | x | y | z | *p*_voxel.FWE_ | k |
| Left supplementary motor cortex (SMC) | -2 | 20 | 52 | < 10^-16^ | 96 |
| Left opercular part of inferior frontal gyrus (OpIFG) | -54 | 14 | 16 | 6.67×10^-16^ | 51 |

**Table S11 Neural correlates of *PTRA* (only for emotional faces) for HC in the task-related phase**

| Positive activity | | | | | |
| --- | --- | --- | --- | --- | --- |
|  | x | y | z | *p*_voxel.FWE_ | *k* |
| Middle occipital gyrus (MOG) | -14 | -98 | -2 | < 10^-16^ | 3231 |
| Right middle cingulate gyrus (MCgG) | 0 | -34 | 32 | < 10^-16^ | 174 |
| Right precuneus (PCu) | 16 | -60 | -60 | < 10^-16^ | 67 |
| Left precuneus (PCu) | -16 | -64 | 28 | < 10^-16^ | 135 |
| Negative activity | | | | | |
|  | x | y | z | *p*_voxel.FWE_ | k |
| Left supplementary motor cortex (SMC) | -4 | 18 | 50 | < 10^-16^ | 435 |
| Left insula (Ins) | -32 | 22 | -2 | < 10^-16^ | 161 |
| Left triangular part of the inferior frontal gyrus (TrIFG) | -38 | 0 | 34 | < 10^-16^ | 402 |
| Left Middle occipital gyrus (MOG) | -30 | -80 | 22 | < 10^-16^ | 77 |
| Right insula (Ins) | 34 | 22 | 0 | < 10^-16^ | 122 |
| Left triangular part of the inferior frontal gyrus (TrIFG) | -44 | 38 | 0 | < 10^-16^ | 118 |
| Right triangular part of the inferior frontal gyrus (TrIFG) | 58 | 24 | 14 | 9.33×10^-14^ | 40 |

**Table S12 Neural correlates of *PTRA* (only for emotional faces) for MDD in the free-viewing phase**

| Positive activity | | | | | |
| --- | --- | --- | --- | --- | --- |
|  | x | y | z | *p*_cluster.FWE *_ | *k* |
| Vermis 6 | 2 | -68 | -14 | 9.48×10^-5^ | 65 |
| Right superior temporal gyrus (STG) | 52 | -10 | 6 | < 10^-16^ | 354 |
| Left superior temporal gyrus (STG) | -40 | -20 | 12 | < 10^-16^ | 363 |
| Left posterior cingulate gyrus (PCgG) | -4 | -44 | 30 | 3.64×10^-4^ | 56 |
| Vermis 8 | 6 | -72 | -42 | 2.52×10^-8^ | 129 |
| Left middle occipital gyrus (MOG) | -20 | -88 | -2 | 2.25×10^-8^ | 130 |
| Left angular gyrus (AnG) | -46 | -62 | 32 | 4.95×10^-4^ | 54 |
| Negative activity | | | | | |
|  | x | y | z | *p*_cluster.FWE *_ | k |
| Left inferior occipital gyrus (IOG) | -36 | -88 | -6 | 9.27×10^-4^ | 82 |
| Right inferior occipital gyrus (IOG) | 36 | -82 | -8 | 8.46×10^-4^ | 50 |
| Left supplementary motor cortex (SMC) | 2 | 16 | 54 | 2.30×10^-4^ | 59 |
| Right opercular part of inferior frontal gyrus (OpIFG) | 50 | 14 | 22 | 1.33×10^-7^ | 115 |
| Right superior frontal gyrus medial segment (MSFG) | 8 | 24 | 44 | 1.98×10^-4^ | 60 |

*Note:* * Statistical maps were collected at a threshold of uncorrected *p* < 0.005, *k* > 50.

**Table S13 Neural correlates of *PTRA* (only for emotional faces) for HC in the free-viewing phase**

| Positive activity | | | | | |
| --- | --- | --- | --- | --- | --- |
|  | x | y | z | *p*_cluster.FWE *_ | *k* |
| Right superior temporal gyrus (STG) | 62 | -16 | 16 | < 10^-16^ | 1088 |
| Left superior temporal gyrus (STG) | -54 | -16 | 12 | < 10^-16^ | 1031 |
| Right postcentral | 32 | -40 | 56 | < 10^-16^ | 587 |
| Left Cerebellum | -34 | -54 | -40 | 7.91×10^-4^ | 51 |
| Vermis 6 | 2 | -68 | -14 | 9.27×10^-4^ | 50 |
| Negative activity | | | | | |
|  | x | y | z | *p*_cluster.FWE *_ | k |
| Right calcarine cortex (Calc) | 14 | -78 | 8 | 3.94×10^-14^ | 260 |
| Right insula (Ins) | 34 | 24 | 4 | 1.68×10^-7^ | 113 |
| Left calcarine cortex (Calc) | -10 | -58 | 16 | 4.52×10^-8^ | 124 |
| Right opercular part of inferior frontal gyrus (OpIFG) | 40 | 10 | 30 | 9.20×10^-8^ | 118 |
| Right inferior occipital gyrus (IOG) | 34 | -86 | -10 | 1.42×10^-8^ | 134 |
| Left insula (Ins) | -30 | 22 | 4 | 7.94×10^-4^ | 51 |
| Left inferior occipital gyrus (IOG) | -32 | -90 | -6 | 6.44×10^-8^ | 121 |

*Note:* * Statistical maps were collected at a threshold of uncorrected *p* < 0.005, *k* > 50.
